# Pseudo-obstruction–inducing *ACTG2^R257C^* alters actin organization and function

**DOI:** 10.1172/jci.insight.140604

**Published:** 2020-08-20

**Authors:** Sohaib Khalid Hashmi, Vasia Barka, Changsong Yang, Sabine Schneider, Tatyana M. Svitkina, Robert O. Heuckeroth

**Affiliations:** 1 Department of Pediatrics, Children’s Hospital of Philadelphia Research Institute, and Perelman School of Medicine at the University of Pennsylvania, Abramson Research Center, Philadelphia, Pennsylvania, USA.; 2 Department of Bioengineering, University of Pennsylvania School of Engineering and Applied Science, Philadelphia, Pennsylvania, USA.; 3 Department of Biology, University of Pennsylvania School of Arts and Sciences, Philadelphia, Pennsylvania, USA.

**Keywords:** Cell Biology, Gastroenterology, Genetic diseases, Muscle

## Abstract

Actin γ 2, smooth muscle (*ACTG2*) *R257C* mutation is the most common genetic cause of visceral myopathy. Individuals with *ACTG2* mutations endure prolonged hospitalizations and surgical interventions, become dependent on intravenous nutrition and bladder catheterization, and often die in childhood. Currently, we understand little about how *ACTG2* mutations cause disease, and there are no mechanism-based treatments. Our goal was to characterize the effects of *ACTG2^R257C^* on actin organization and function in visceral smooth muscle cells. We overexpressed *ACTG2^WT^* or *ACTG2^R257C^* in primary human intestinal smooth muscle cells (HISMCs) and performed detailed quantitative analyses to examine effects of ACTG2^R257C^ on (a) actin filament formation and subcellular localization, (b) actin-dependent HISMC functions, and (c) smooth muscle contractile gene expression. ACTG2^R257C^ resulted in 41% fewer, 13% thinner, 33% shorter, and 40% less branched ACTG2 filament bundles compared with ACTG2^WT^. Curiously, total F-actin probed by phalloidin and a pan-actin antibody was unchanged between *ACTG2^WT^*- and *ACTG2^R257C^*-expressing HISMCs, as was ultrastructural F-actin organization. *ACTG2^R257C^*-expressing HISMCs contracted collagen gels similar to *ACTG2^WT^*-expressing HISMCs but spread 21% more and were 11% more migratory. In conclusion, *ACTG2^R257C^* profoundly affects ACTG2 filament bundle structure, without altering global actin cytoskeleton in HISMCs.

## Abstract 2

A mutation associated with visceral myopathy profoundly disrupts actin gamma 2, smooth muscle (ACTG2) filament bundles without changing the global actin cytoskeleton in visceral smooth muscle cells.

## Introduction

Heterozygous point mutations in actin γ 2, smooth muscle (*ACTG2*) are the primary cause of visceral myopathy (1, 2). Visceral myopathies are characterized by smooth muscle cell (SMC) weakness and bowel, bladder, and uterine dysfunction. The most severe manifestation is megacystis, microcolon, intestinal hypoperistalsis syndrome (MMIHS), where gut and bladder dysfunction begin before birth, intravenous nutrition and bladder catheters are common, and childhood survival is 19.7% (3). Visceral myopathy–associated bowel dysfunction is called chronic intestinal pseudo-obstruction (CIPO). Remarkably, some people with pathogenic *ACTG2* mutations survive to adulthood without intravenous nutrition and have disease recognized later in life, whereas others with the same *ACTG2* mutation are seriously ill in childhood (2, 4, 5). Nonetheless, *ACTG2*-associated visceral myopathy typically leads to prolonged hospitalization, repeated surgery, nutritional deficiencies, and serious complications from intravenous nutrition. Current therapies are largely supportive and ineffective. Disease mechanisms remain poorly defined, making it challenging to develop new treatments.

Actin, the most abundant protein in eukaryotic cells, has 6 isoforms: skeletal muscle (*ACTA1*), cardiac muscle (*ACTC1*), α smooth muscle (*ACTA2*), γ smooth muscle (*ACTG2*), β cytoplasmic (*ACTB*), and γ cytoplasmic (*ACTG1*). These isoforms share ≥93% amino acid identity but have distinct functions (6) and subcellular localization. Many actin functions rely on polymerization of monomeric G-actin to filamentous F-actin, and some isoforms copolymerize (7). Diverse actin functions include roles in contractility, migration, spreading, signal transduction, and transcriptional regulation. When 1 isoform is missing, another may be upregulated. However, actin isoforms only partially compensate for each other (6). For example, expressing *Actg2* in cardiac muscle of *Actc1^–/–^* mice partially rescues function, but hearts are hypodynamic and hypertrophic (8). The reasons for partial compensation remain unknown.

SMC contraction depends on F-actin–myosin interactions, as in striated muscle, but SMCs lack sarcomeres. Instead, contractile structures oriented obliquely to the SMC long axis, anchored at dense bodies and plaques, allow SMCs to maintain maximum actin-myosin overlap and generate force over a wide range of cell lengths, unlike in striated muscle (9). In addition to contractile apparatus plasticity, SMCs have remarkable differentiation plasticity and can transition from contractile to synthetic phenotypes. Contractile SMCs are spindle shaped and express high levels of contractile genes. Synthetic SMCs spread on surfaces, migrate, produce more extracellular matrix (ECM), and downregulate contractile genes. This phenotypic switch in response to mechanobiochemical factors can be beneficial, facilitating tissue regeneration after injury (10), or pathologic, causing fibrosis in inflammatory bowel disease (11) and vascular disease (12).

It is not well understood how *ACTG2* mutations cause visceral myopathy. *ACTG2* is the predominant actin isoform in visceral SMCs, but these cells also express *ACTA2* and cytoplasmic actins, *ACTB* and *ACTG1*, with distinct functions. For example, knockdown of *ACTA2* in vascular SMCs impairs contractility, but *ACTG2* knockdown does not (13). Furthermore, aneurysm-causing *ACTA2* mutations disrupt contractile apparatus interactions with ECM elastin (14). Consistent with distinct functions, ACTG2- and ACTA2-stained F-actin appear in different locations in vascular SMCs (13). These observations led to the hypothesis that ACTA2 is critical for stable contractile filaments, while ACTG2 is more important for dynamic submembranous actin that polymerizes in response to contractile stimuli (14, 15). This submembranous network links ECM to cytoskeleton, enhancing membrane rigidity as tension is generated via contractile actin-myosin interactions (15, 16). Actin cytoskeleton polymerization dynamics near the membrane are also critical for development of tension and transmission of generated force from the contractile apparatus to the ECM and to neighboring cells. For these reasons, ACTG2 may have roles in cell migration, spreading, and resistance to passive stretch, with less important roles in active contraction. However, our knowledge of normal ACTG2 physiology remains incomplete, and we need to know more to understand how mutations cause disease.

Twenty-five distinct *ACTG2* mutations are implicated in MMIHS/CIPO. The most common is arginine 257 to cysteine (*R257C*) (11% of MMIHS/CIPO) (1). These missense mutations often arise de novo, causing disease in heterozygotes, suggesting dominant-negative mechanisms because loss of 1 *ACTG2* allele did not cause disease (4). Prior studies of overexpressed ACTG2 mutants (R148S, R178C, R178L, R178H, R40C, and R63Q) showed inefficient incorporation into F-actin and reduced collagen gel contraction by cells (17–19). However, these studies used cancer cells that do not normally express *ACTG2* and may lack visceral SMC components that influence *ACTG2* expression or function. Furthermore, quantitative analyses were limited.

Here, we characterize *ACTG2*^R257C^** using human intestinal SMCs (HISMCs) that normally express *ACTG2*. Although HISMCs expressing untagged *ACTG2*^R257C^** had a normal ability to radially contract collagen gels, *ACTG2*^R257C^-expressing cells spread more on surfaces and migrated faster than *ACTG2^WT^*-expressing HISMCs. Furthermore, V5-tagged ACTG2^R257C^ formed filament bundles less efficiently than V5-ACTG2^WT^, producing V5-ACTG2^R257C^ F-actin bundles that were thinner, shorter, and less branched than ACTG2^WT^ V5-ACTG2 F-actin bundles. Intriguingly, total F-actin visualized with phalloidin or by electron microscopy had normal abundance and structural characteristics in untagged *ACTG2*^R257C^**-expressing HISMCs, consistent with distinct pools of ACTG2 and “other” actin filaments. We did not, however, see preferential localization of ACTG2 in specific cell regions and found ACTG2^WT^ and ACTG2^R257C^ in the same filament bundles. Collectively, these observations suggest that ACTG2^R257C^ induces a more motile, less contractile phenotype (20, 21), without profound disruption of overall actin cytoskeletal networks in HISMCs.

## Results

### Modeling suggests little impact of ACTG2^R257C^ on actin conformation

ACTG2^R257^ is predicted to be important for interstrand contacts for F-actin polymerization or bundling (22). Since there is no structural data for ACTG2, we used Modeller (23) with a rabbit ACTA1 template to model the impact of ACTG2^R257C^. G-actin ACTG2^R257C^ was modeled bound to ADP. F-actin ACTG2^R257C^ was modeled using formin FH2–bound actin with ATP, since FH2-bound actin closely resembles F-actin (24). ACTG2^R257C^ aligned closely with ACTG2^WT^ protein, without major differences in ribbon structure (Supplemental Figure 1; supplemental material available online with this article; https://doi.org/10.1172/jci.insight.140604DS1). Since the cysteine R group is shorter than the arginine R group, we looked for differences in steric clashes within the ACTG2^WT^ and ACTG2^R257C^ mutant protein structures. Using all-atom contact analysis in MolProbity (25), we determined that the normal F-actin molecule has a steric clash between R257 and F223, with an overlap of 0.6 Å (any overlap greater than 0.4 is considered a clash). Such clash is not present in the ACTG2^R257C^ F-actin and was not seen in either ACTG2^WT^ or ACTG2^R257C^ G-actin structures. The absence of this steric clash in the ACTG2^R257C^ mutant F-actin molecule may have implications for filament structure. Ramachandran plots to visualize energetically allowed conformations of residues in secondary structures showed few differences (Supplemental Figure 2). ACTG2^WT^ and ACTG2^R257C^ have similar allowed and favorable dihedral angles and minor differences in outlier residues with unusual dihedral angles. Finally, unlike R257, C257 might interact with C218 (Supplemental Figure 3), but thermodynamics of potential disulfide bond formation between these cysteine residues are unknown and distance is approximately 3.2-fold longer than a disulfide bond in these models (26). Collectively, these observations suggest that the effects of ACTG2^R257C^ on ACTG2 secondary structure are not dramatic, although molecular dynamics simulations or modeling with F-actin–binding proteins, such as tropomyosin, might show additional problems.

### HISMCs expressing ACTG2^R257C^ and ACTG2^WT^ proteins have similar abundance of smooth muscle contractile genes

**Figure 1:**
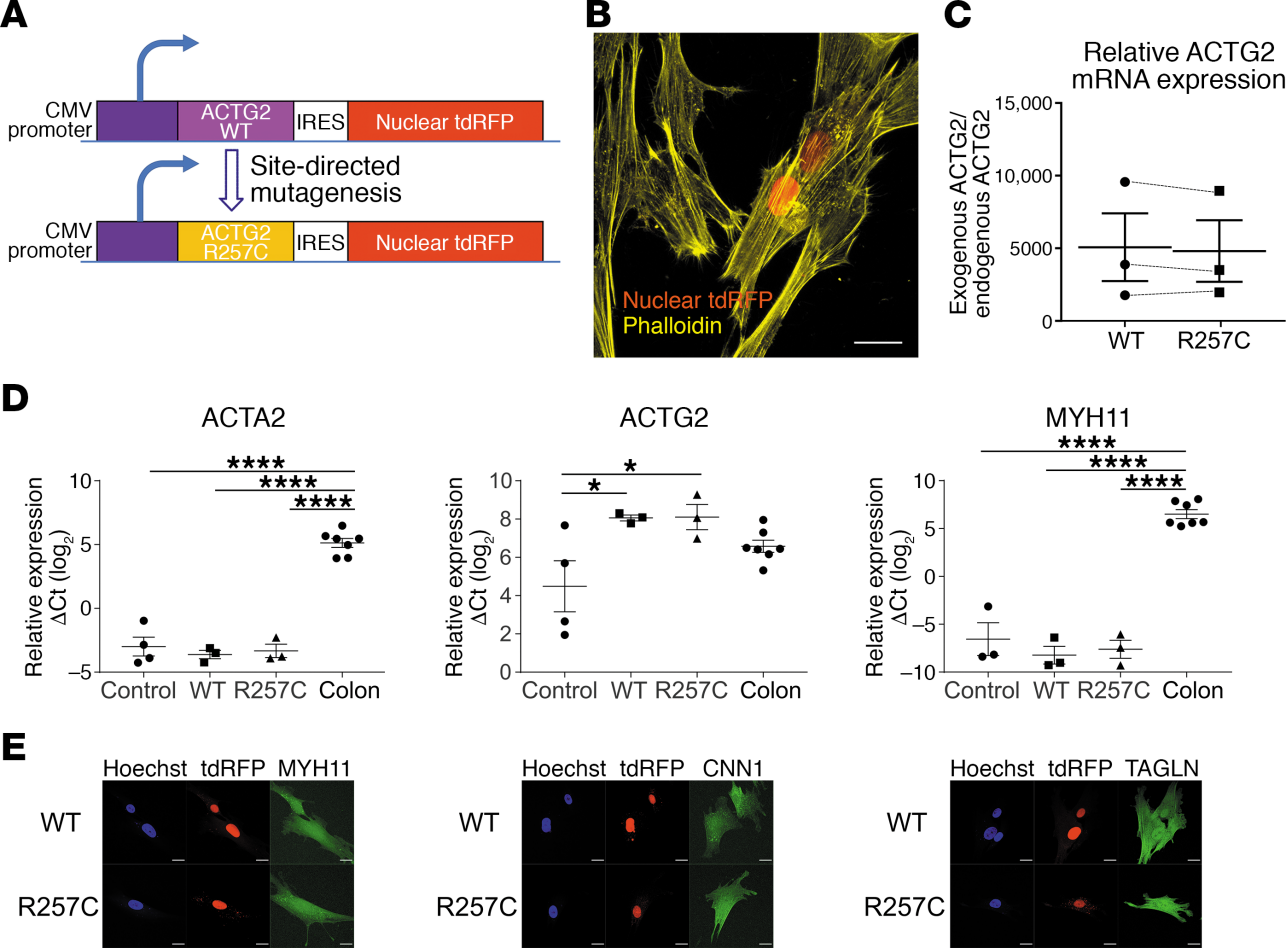
Overexpression constructs and HISMC validation. (**A**) CMV promoter drives *ACTG2* and *ntdRFP* expression from the same mRNA. (**B**) Exogenous *ACTG2*-expressing HISMCs were identified by ntdRFP (red) coexpression. F-actin, phalloidin (yellow) (scale bar: 20 μm). The average transfection efficiency (percentage of ntdRFP^+^ cells) is approximately 15%. (**C**) Exogenous/endogenous *ACTG2* ratios (qRT-PCR) were similar for *ACTG2^WT^* and *ACTG2^R257C^* (*n* = 3; paired Student’s *t* test, *P* = 0.3893). (**D**) mRNA levels for smooth muscle contractile genes *ACTA2*, *ACTG2*, and *MYH11* were similar in *ACTG2^WT^-* and *ACTG2^R257C^*-expressing HISMCs. *ACTG2* levels were similar in transfected HISMCs and freshly isolated human colon smooth muscle. *ACTA2* and *MYH11* mRNAs were less abundant in HISMCs compared with freshly isolated smooth muscle. *YWHAZ* was used as normalization control (sample size: *n* = 4 for control HISMCs, *n* = 3 each for ACTG2^WT^- or ACTG2^R257C^-expressing HISMCs, and *n* = 7 for human colon smooth muscle). *P < 0.05, ****P < 0.0001, 1-way ANOVA with Tukey’s multiple comparison test. (**E**) Confocal *Z*-stacks (maximum intensity projections) of *ACTG2^WT^*- or *ACTG2^R257C^*-expressing HISMCs were stained for smooth muscle markers (MYH11, CNN1, and TAGLN) (scale bar: 20 μm). Abundance of these proteins appeared similar in *ACTG2^WT^*- and *ACTG2^R257C^*-expressing HISMCs 48 hours after transfection. Images are representative of 3 independent experiments.

Reduced contractile gene expression was reported in bowels from individuals with MMIHS/CIPO (27). To test the hypothesis that ACTG2^R257C^ alters expression of contractile genes, we transfected HISMCs to overexpress untagged *ACTG2^WT^* or *ACTG2^R257C^*. An internal ribosome entry site (IRES) 3′ to *ACTG2* followed by nuclear-localized tandem dimer red fluorescent protein (ntdRFP) allowed ACTG2 and ntdRFP translation from 1 mRNA (Figure 1A). Cells expressing *ACTG2* were therefore readily isolated based on ntdRFP fluorescence (Figure 1B and Supplemental Figure 4). *ACTG2*^WT^** and *ACTG2*^R257C^** mRNA levels were approximately 5000-fold higher than endogenous *ACTG2* in transfected HISMCs (Figure 1C). As anticipated, transfected *ACTG2^WT^* and *ACTG2^R257C^* mRNA levels were similar (*ACTG2^WT^*, 5072 ± 2330–fold; *ACTG2^R257C^*, 4808 ± 2120–fold higher than endogenous *ACTG2*; *P* = 0.3893). Interestingly, total *ACTG2* mRNA levels vary by only approximately 12-fold between transfected and untransfected cells, suggesting that exogenous ACTG2 potently repressed endogenous *ACTG2* mRNA (Figure 1D). Furthermore, *ACTG2* mRNA levels in transfected and untransfected HISMCs were within approximately 2 cycles (ΔC_t_ — higher values indicate more mRNA) of *ACTG2* mRNA levels in freshly isolated human colon smooth muscle (ΔC_t_ *ACTG2*: untransfected HISMCs, 4.490 ± 1.335; freshly isolated human colon smooth muscle, 6.583 ± 0.317; *ACTG2^WT^*, 8.06 ± 0.153; *ACTG2^R257C^*, 8.104 ± 6.583).

In contrast to endogenous *ACTG2*, mRNA levels for other major SMC contractile genes (*ACTA2* and *MYH11*) were not altered by exogenous *ACTG2^WT^* or *ACTG2^R257C^* expression (ΔC_t_ *ACTA2*: *ACTG2^WT^*, –3.616 ± 0.325; *ACTG2^R257C^*, –3.332 ± 0.521; untransfected control, –2.995 ± 0.738; *MYH11*: *ACTG2^WT^*, –8.233 ± 0.920; *ACTG2^R257C^*, –7.613 ± 0.938; untransfected control, –6.566 ± 1.716) (Figure 1D). However, transfected and untransfected cultured HISMCs had significantly less *ACTA2* and *MYH11* mRNA than freshly isolated smooth muscle (human colon smooth muscle ΔC_t_: *ACTA2*, 5.131 ± 0.3513; *MYH11*, 6.505 ± 0.4658), likely from transition in culture to synthetic phenotype. Abundance of SMC contractile proteins MYH11, CNN1, and TAGLN also appeared similar in *ACTG2^WT^*- and *ACTG2^R257C^*-expressing HISMCs based on immunohistochemistry (Figure 1E). Consistent with these results, nuclear-to-cytoplasmic ratio of myocardin-related transcription factor A (MRTF-A), a major regulator of contractile gene expression, was equivalent in *ACTG2^WT^*- and *ACTG2^R257C^*-expressing cells (Supplemental Figure 5).

### ACTG2^R257C^ is poorly incorporated into actin filament bundles and alters F-actin bundle structure

**Figure 2:**
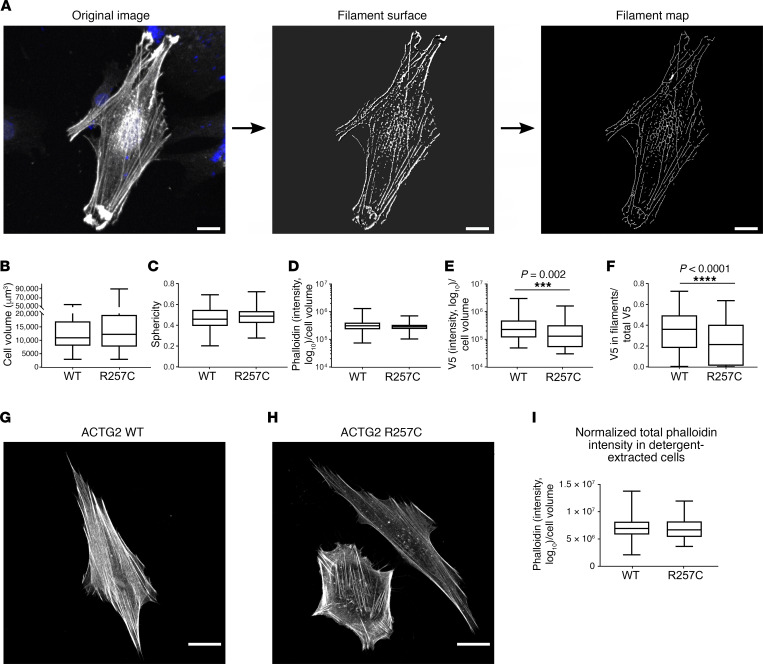
Quantitative analysis of V5 antibody– and phalloidin-stained HISMCs expressing V5-ACTG2^WT^ and V5-ACTG2^R257C^ proteins. (**A**) Imaris analysis pipeline. Whole HISMCs were manually outlined based on V5 staining to generate surfaces. V5 and phalloidin staining of filaments was used to generate surfaces corresponding to F-actin bundles. These surfaces were used to generate filament network maps (scale bar: 20 μm). *V5-ACTG2^WT^*– or *V5-ACTG2^R257C^*–expressing HISMCs were compared. (**B**) Cell volume (Mann-Whitney test, *P* = 0.2875) and (**C**) cell sphericity (Mann-Whitney test, *P* = 0.5947) were equivalent. (**D**) Total phalloidin level (normalized to cell volume) was equivalent in *ACTG2^WT^*- and *ACTG2^R257C^*-expressing HISMCs (median [interquartile range], *ACTG2^WT^* 309715 AU [174142 AU], *ACTG2^R257C^* 286576 AU [98657 AU]; Mann-Whitney test, *P* = 0.0688). (**E**) Total V5 level (normalized to cell volume) was lower in *ACTG2^R257C^*-expressing HISMCs compared with *ACTG2^WT^*-expressing HISMCs (median [interquartile range], *ACTG2^WT^* 226,527 AU [366,604 AU], *ACTG2^R257C^* 129,985 AU [271,860 AU]; Mann-Whitney test, *P* = 0.002). (**F**) V5 in filaments/total V5 was lower in *ACTG2^R257C^*-expressing HISMCs compared with *ACTG2^WT^*-expressing HISMCs (median [interquartile range], *ACTG2^WT^* 0.361 [0.314], *ACTG2^R257C^* 0.214 [0.394]; Mann-Whitney test, *P* < 0.0001) (**B–F**: *n* = 3; ACTG2^WT^, 97 cells; ACTG2^R257C^, 84 cells). HISMCs transfected with (**G**) *ACTG2^WT^-IRES-ntdRFP* or (**H**) *ACTG2^R257C^-IRES-ntdRFP* detergent extracted with 0.5% Triton X-100. Representative confocal *Z*-stacks with “sum of slices” projection (scale bar: 20 μm). (**I**) Phalloidin intensity was equivalent in *ACTG2^WT^*- and *ACTG2^R257C^*-expressing HISMCs (**G–I**: *n* = 3; ACTG2^WT^, 83 cells; *ACTG2^R257C^*, 78 cells; median [interquartile range], *ACTG2^WT^* 6,934,687 AU [2,366,386 AU], *ACTG2^R257C^* 6,685,969 AU [284,8424 AU]; Mann-Whitney test; *P* = 0.3885). Quantitative data are represented as box-and-whisker plots. The box extends from 25th to 75th percentile, and median is indicated by horizontal line. Whiskers represent maximum and minimum values.

We wanted to directly observe if ACTG2^R257C^ disrupted ACTG2-containing actin structures in HISMCs. Since commercially available antibodies do not reliably distinguish ACTG2 from other actin isoforms, we designed N-terminal V5-tagged *ACTG2* overexpression vectors to track ACTG2^WT^ and mutant proteins. We used V5 antibody to localize exogenous V5-ACTG2 and phalloidin to stain all F-actin. In contrast to other *ACTG2* variants reported (17–19), we did not see obvious defects in actin filaments in *V5*-*ACTG2^R257C^*–expressing HISMCs after staining with V5 antibody or phalloidin. However, we identified major defects by performing detailed quantitative analysis of cell shape and F-actin content and organization in 3D images of *V5*-*ACTG2^WT^*– or *V5*-*ACTG2^R257C^*–expressing HISMCs (Figure 2A).

#### Cell-level descriptors.

*V5*-*ACTG2*^WT^**– and *V5-ACTG2^R257C^*–expressing HISMCs had similar cell volume and sphericity (Figure 2, B and C), suggesting that mutant protein did not cause major disruption to actin structures that control cell shape. Total phalloidin staining intensity was also equivalent in *ACTG2^WT^* and *ACTG2^R257C^* mutant *V5-ACTG2* expressing HISMCs (Figure 2D). In contrast, total V5 intensity was 43% lower for ACTG2^R257C^ protein than for ACTG2^WT^ (Figure 2E). The fraction of V5-ACTG2^R257C^ in filaments was also 41% reduced compared with ACTG2^WT^ (Figure 2F). Given differences between V5 antibody and phalloidin data, we repeated phalloidin analyses on HISMCs expressing untagged *ACTG2-IRES-ntdRFP*. Phalloidin intensity was still similar in *ACTG2^WT^*- and *ACTG2^R257C^*-expressing cells (Figure 2, G–I). Since mRNA levels were comparable for *ACTG2^WT^* and *ACTG2^R257C^* (Figure 1D), these data suggest that ACTG2^R257C^ protein has reduced stability and abnormal incorporation into F-actin.

#### Filament network descriptors.

**Figure 3:**
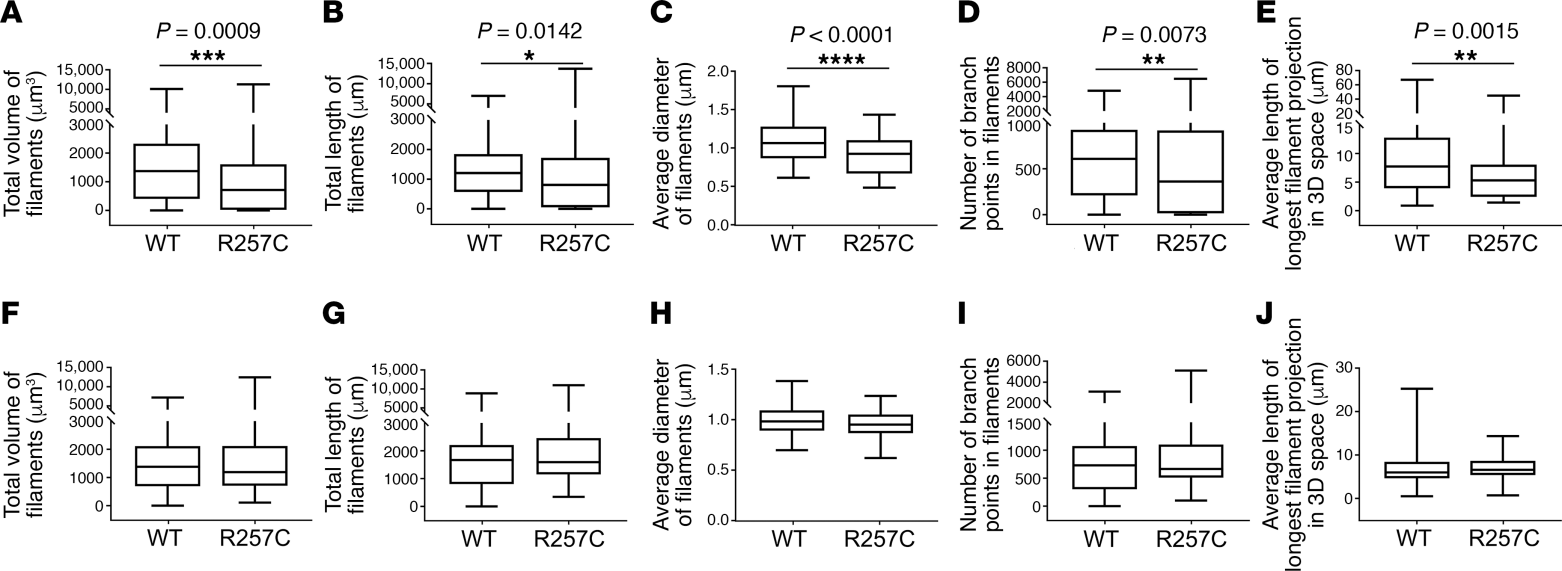
V5-ACTG2^R257C^ decreased V5–stained F-actin bundle volume, length, diameter, and branching. HISMCs expressing *V5-ACTG2^WT^* or *V5-ACTG2^R257C^* were compared using filament maps (Figure 2A). (**A**) V5-tagged filament volume was lower in *ACTG2^R257C^*-expressing HISMCs compared with *ACTG2^WT^*-expressing HISMCs (median [interquartile range], *ACTG2^WT^*, 1370 μm^3^ (1926.7 μm^3^); *ACTG2^R257C^*, 711.9 μm^3^ (1592.9 μm^3^); Mann-Whitney test, *P* = 0.009). (**B**) Total V5-tagged filament length was lower in *ACTG2^R257C^*-expressing HISMCs compared with *ACTG2^WT^*-expressing HISMCs (median [interquartile range], *ACTG2^WT^* 1211 μm [1279.8 μm], *ACTG2^R257C^* 807.36 μm [1669.2 μm]; Mann-Whitney test, *P* = 0.0142). (**C**) Average V5-tagged filament diameter was smaller in ACTG2^R257C^-expressing HISMCs compared with *ACTG2^WT^*-expressing HISMCs (median [interquartile range], *ACTG2^WT^* 1.065 μm [0.4138 μm], *ACTG2^R257C^* 0.9244 μm [0.4349 μm]; Mann-Whitney test, *P* < 0.0001). (**D**) The number of V5-tagged filament branch points was lower in *ACTG2^R257C^*-expressing HISMCs compared with *ACTG2^WT^*-expressing HISMCs (median [interquartile range], *ACTG2^WT^* 607 [715.8], *ACTG2^R257C^* 361.5 [902.5]; Mann-Whitney test, *P* = 0.0073). (**E**) Average length of longest V5-tagged filament projections in 3D space was shorter in *ACTG2^R257C^*-expressing HISMCs compared with *ACTG2^WT^*-expressing HISMCs (median [interquartile range], *ACTG2^WT^* 7.742 μm [8.872 μm], *ACTG2^R257C^* 5.306 μm [5.655 μm]; Mann-Whitney test, *P* = 0.0015). All of these parameters were equivalent for phalloidin-labeled filaments in *ACTG2^WT^*- and *ACTG2^R257C^*-expressing HISMCs (**F–J**). Quantitative data are represented as box-and-whisker plots. The box extends from 25th to 75th percentile, and median is indicated by horizontal line. Whiskers represent maximum and minimum values. *n* = 3; ACTG2^WT^, 97 cells; ACTG2^R257C^, 84 cells.

We next pursued quantitative analysis of actin filament bundles in HISMCs using Imaris to create 3D surfaces surrounding V5- and phalloidin-stained F-actin (Figure 2A and Supplemental Video 1). Based on V5 staining, cells expressing *ACTG2^R257C^* had 48% lower total actin filament volume than cells expressing *V5-ACTG2^WT^* (Figure 3A). Mutant V5-ACTG2–containing filament bundles also had 33% lower total length (Figure 3B), 13% smaller average diameter (Figure 3C), 40% less branching (Figure 3D), and a 31% reduction in length for the longest filament projection in 3D space (Figure 3E). Interestingly, analysis of phalloidin-stained F-actin bundles in these same cells showed that expression of *V5-ACTG2^R257C^* did not change any of these parameters (Figure 3, F–J). There was also no difference in straightness of V5- or phalloidin-labeled filament bundles (Supplemental Figure 6). Thus, ACTG2^R257C^ protein was incorporated into fewer, shorter, thinner, and less branched ACTG2-containing F-actin bundles compared with ACTG2^WT^, but overall organization of F-actin, as indicated by phalloidin labeling, was equivalent in *ACTG2^WT^* and *ACTG2^R257C^*-expressing HISMCs, suggesting that other actin isoforms compensated for inferior incorporation of ACTG2^R257C^ into actin structures. In contrast to vascular SMCs (13), V5-tagged ACTG2^WT^ or ACTG2^R257C^ did not appear to be restricted to the center of the cell but instead overlapped phalloidin staining equivalently in central and peripheral regions (Supplemental Figure 7).

### *ACTG2R*^257C^ and ACTG2^WT^ are in the same F-actin bundles

To determine if ACTG2^WT^ and ACTG2^R257C^ proteins were incorporated into the same F-actin bundles, we cotransfected HISMCs to express N-terminal FLAG- or V5-tagged ACTG2, permitting simultaneous visualization of ACTG2^WT^ and mutant protein. Because FLAG and V5 might differentially affect ACTG2 function, HISMCs were transfected with either *FLAG-ACTG2^R257C^-IRES-ntdRFP* plus *V5-ACTG2^WT^-IRES-nEGFP* or with *FLAG-ACTG2^WT^-IRES-ntdRFP* plus *V5-ACTG2^R257C^-IRES-nEGFP*. We found *ACTG2*^WT^ and ACTG2^R257C^ in the same filament bundles throughout the cell (Supplemental Figure 8, A and B). While most ACTG2 was in filaments, we also saw ACTG2^WT^ and mutant ACTG2 in spots that could be protein aggregates. Consistent with phalloidin data, V5-ACTG2^R257C^ was as likely as ACTG2^WT^ to be found in filament bundles in all regions of the cell.

### *ACTG2R*^257C^ does not cause obvious ultrastructural defects in F-actin

To visualize actin filaments at higher resolution, HISMCs expressing *ACTG2^WT^-IRES-ntdRFP* or *ACTG2^R257C^-IRES-ntdRFP* were examined by platinum replica electron microscopy (28). We examined F-actin–containing stress fibers in the densest cortical region midway between the nucleus and the leading edge (Supplemental Figure 9A) and F-actin in lamella and lamellipodia at the leading edge of HISMCs (Supplemental Figure 9B). We did not observe any striking systematic differences in F-actin organization or filament appearance between *ACTG2^WT^*- and *ACTG2^R257C^*-expressing HISMCs.

### *ACTG2*^R257C^ is not efficiently incorporated into F-actin

**Figure 4:**
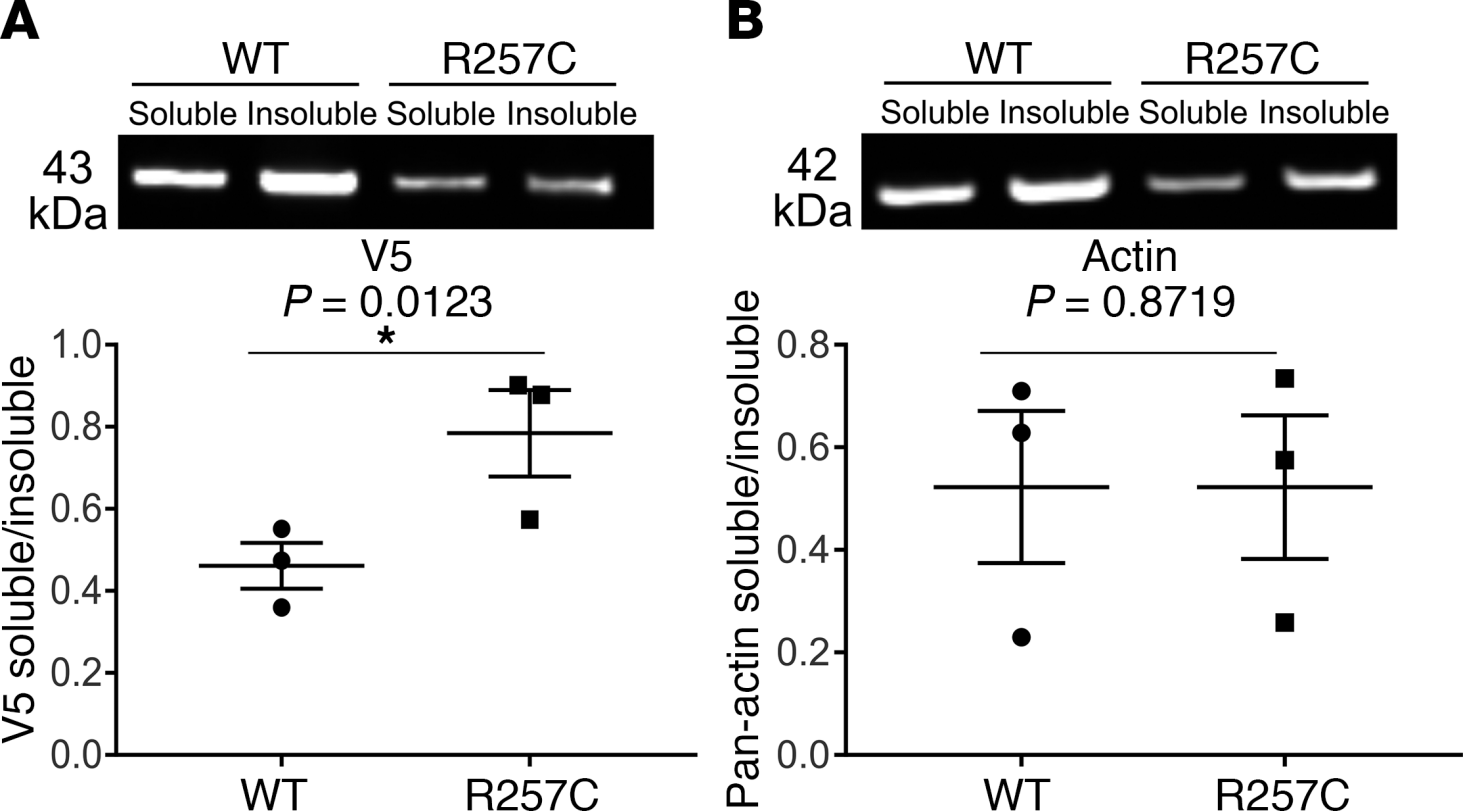
ACTG2^R257C^ is inefficiently incorporated into F-actin. Triton X-100–insoluble cytoskeletal assay was performed to extract soluble and insoluble fractions of sorted HISMCs transfected with *V5-ACTG2*^WT^*-IRES-nEGFP* or *V5-ACTG2*^R257C^*-IRES-nEGFP*. Western blot used antibodies against (**A**) V5-tagged ACTG2 and (**B**) pan-actin. *ACTG2^R257C^*-expressing HISMCs had a higher proportion of V5-ACTG2 in soluble versus insoluble fractions (*n* = 3; *ACTG2^WT^*, 0.4614 ± 0.05561; *ACTG2^R257C^*, 0.7842 ± 0.1053; ratio paired Student’s *t* test, *P* = 0.0123). The pan-actin soluble/insoluble ratio did not differ between *ACTG2^WT^-* and *ACTG2^R257C^*-expressing HISMCs (*n* = 3; *ACTG2^WT^*, 0.5225 ± 0.0.1483; *ACTG2^R257C^*, 0.5226 ± 0.1400; ratio paired Student’s *t* test, *P* = 0.8719).

To confirm imaging data, we separated G-actin from F-actin using Triton X-100 extraction. HISMCs transfected with V5-*ACTG2*^WT^**- or *ACTG2^R257C^*-IRES-nEGFP were sorted to isolate EGFP^+^ cells 48 hours after transfection, cultured 24 additional hours, and then permeabilized with 1% Triton X-100–containing lysis buffer. After collecting the Triton X-100–soluble proteins, the remaining insoluble cytoskeleton-associated fraction was solubilized with 1% SDS–containing lysis buffer. Western blot showed a 41% increase in the soluble/insoluble ratio for V5-ACTG2^R257C^ compared with V5-ACTG2^WT^ protein (Figure 4A). In contrast, pan-actin antibody demonstrated no significant difference in the soluble/insoluble actin ratio (Figure 4B), consistent with imaging analyses.

### *ACTG2R257C*-expressing HISMCs spread more and migrate faster

We evaluated the functional consequences of *ACTG2*^R257C^** expression in HISMCs by collagen gel contraction, cell migration, and cell spreading. Each of these processes requires force generation by the actin cytoskeleton.

#### Collagen gel contraction assay.

**Figure 5:**
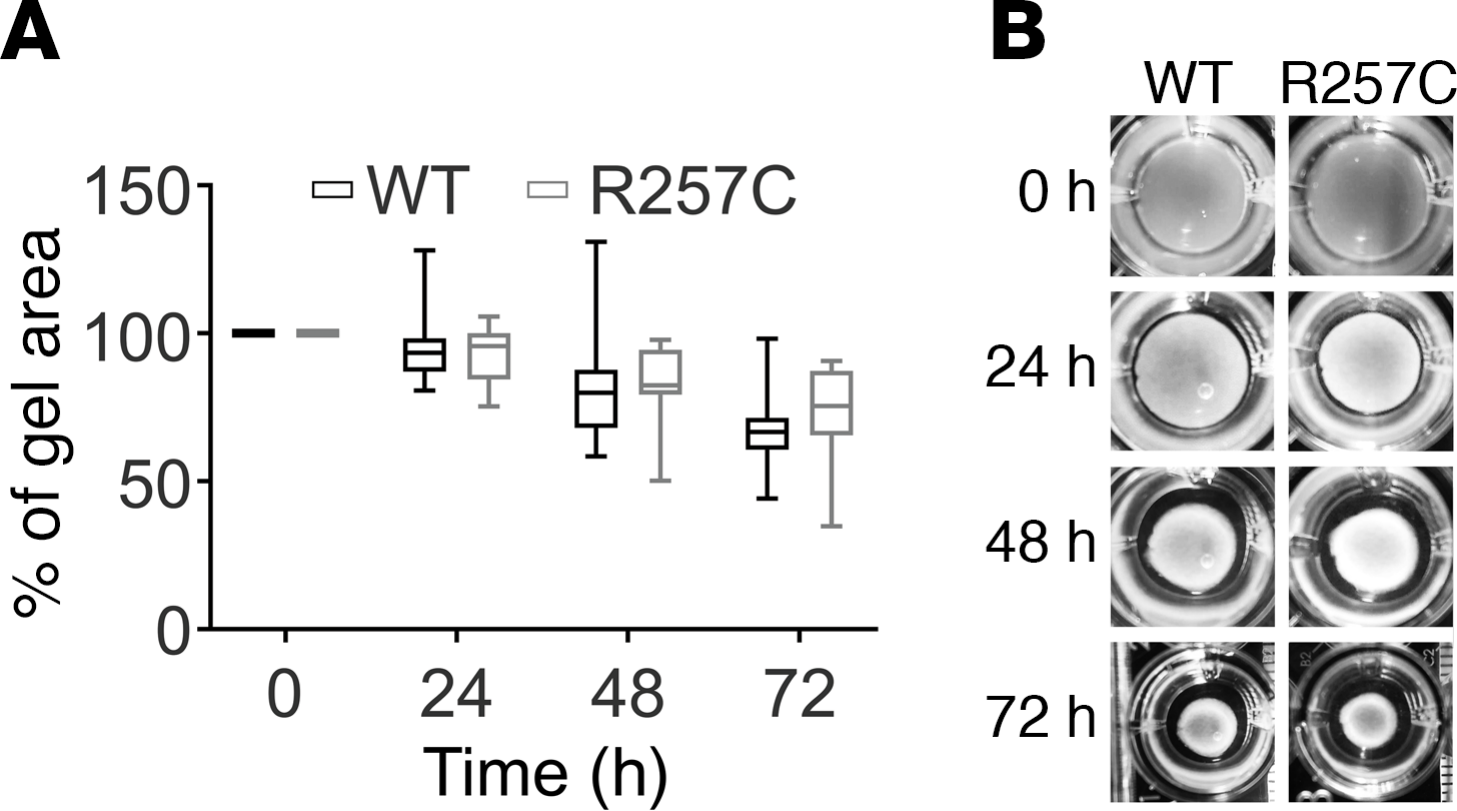
ACTG2^R257C^ does not affect radial collagen gel contraction. (**A**) Collagen gel contraction was similar in *ACTG2^WT^*- or *ACTG2^R257C^*-expressing HISMCs (*n* = 6 independent experiments, ≥2 wells for each group per experiment, Mann-Whitney test; 24 hours: *ACTG2^WT^*, 93.49% [11.23%]; *ACTG2^R257C^*, 95.65% [15.53%]; *P* = 0.8907; 48 hours: *ACTG2^WT^*, 79.96% [19.67%]; *ACTG2^R257C^*, 82.45% [15.12%]; *P* = 0.3996; 72 hours: *ACTG2^WT^*, 66.67% [10.66%]; *ACTG2^R257C^*, 75.41% [21.83%]; *P* = 0.07). Quantitative data are represented as box-and-whisker plots. The box extends from the 25th to 75th percentile, and the median is indicated by the horizontal line. Whiskers represent maximum and minimum values. Median [interquartile range] values are presented in legend. (**B**) Representative images at 0, 24, 48, and 72 hours.

Since *ACTG2* mutations cause visceral SMC weakness, we analyzed contraction of free-floating collagen gels by embedded HISMCs (Figure 5A). *ACTG2*^WT^*-* and *ACTG2^R257C^*-expressing HISMCs reduced collagen gel cross-sectional area equivalently at 24, 48, or 72 hours (Figure 5B).

#### Cell migration assay.

**Figure 6:**
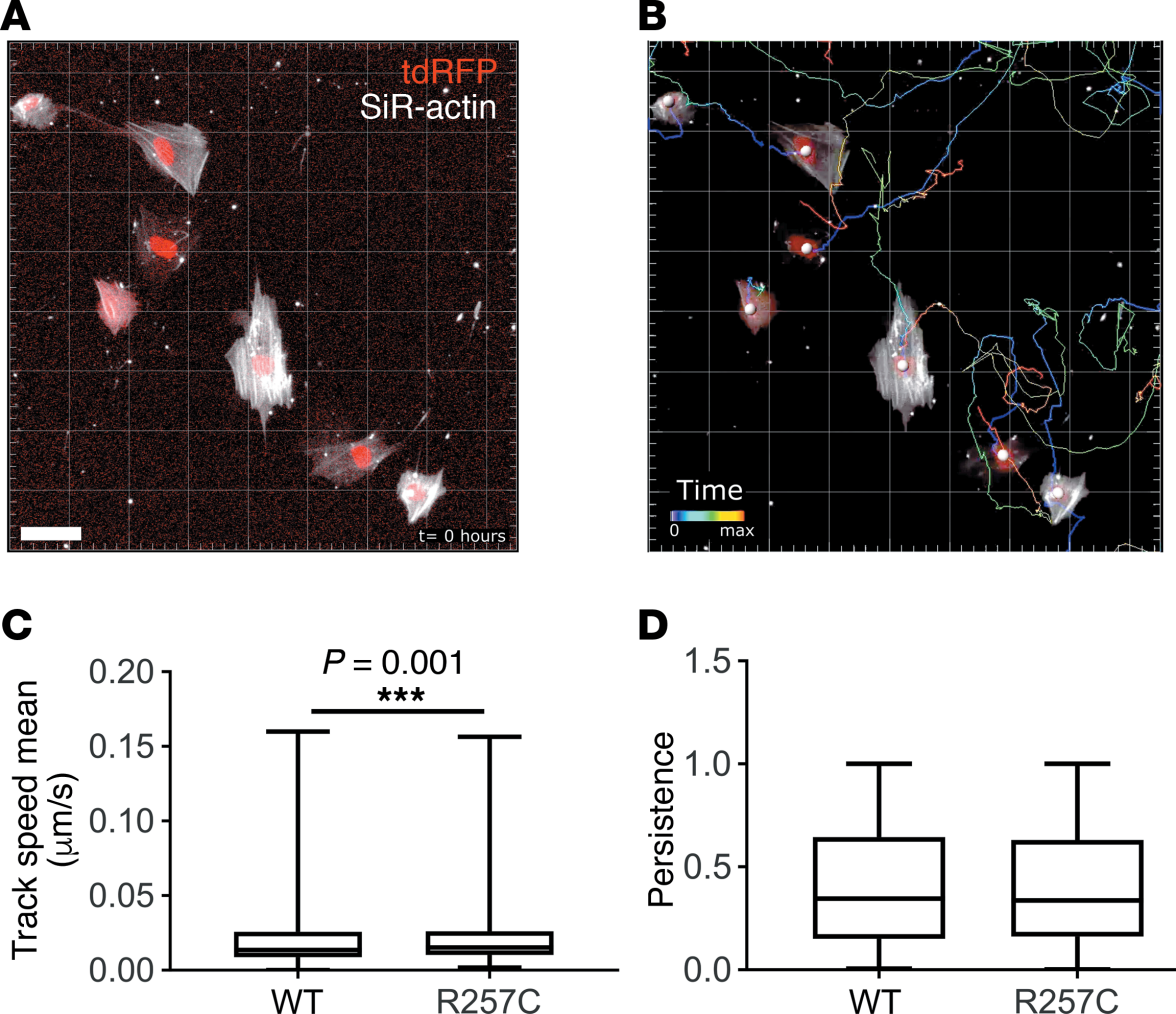
ACTG2^R257C^ enhances HISMC migratory behavior. (**A**) Representative image showing HISMCs at *t* = 0 (scale bar: 50 μm). (**B**) Image from **A** after processing with superimposed time trace for 12 hours. Nuclei identified by *Spots* algorithm in Imaris are marked by gray spheres. (**C**) Mean track speed was faster (11%) for *ACTG2^R257C^*-expressing HISMCs compared with *ACTG2^WT^*-expressing HISMCs (median [interquartile range], *ACTG2^WT^*, 0.01364 μm/s [0.01694 μm/s], *ACTG2^R257C^*, 0.01525 μm/s [0.01587 μm/s]; Mann-Whitney test, *P* = 0.001). (**D**) Persistence (displacement/distance) was similar in *ACTG2^WT^*- and *ACTG2^R257C^*-expressing HISMCs (median [interquartile range], *ACTG2^WT^*, 0.3459 [0.4936]; *ACTG2^R257C^*, 0.3368 [0.4692]; Mann-Whitney test, *P* = 0.4864). *n* = 4 independent experiments; ACTG2^WT^, 952 cells; ACTG2^R257C^, 1192 cells. Quantitative data are represented as box-and-whisker plots. The box extends from 25th to 75th percentile, and median is indicated by horizontal line. Whiskers represent maximum and minimum values.

To determine if *ACTG2*^R257C^** alters migratory activity of HISMCs, cultured *ACTG2*^WT^**- or *ACTG2^R257C^*-IRES-ntdRFP–expressing cells were followed by time-lapse microscopy (Figure 6A). Using nuclear tdRFP, we tracked cells for 12 hours (Figure 6B). *ACTG2*^R257C^**-expressing HISMCs moved approximately 11% faster than *ACTG2*^WT^**-expressing HISMCs (Figure 6C), but persistence was equivalent (Figure 6D).

#### Cell spreading assay.

**Figure 7:**
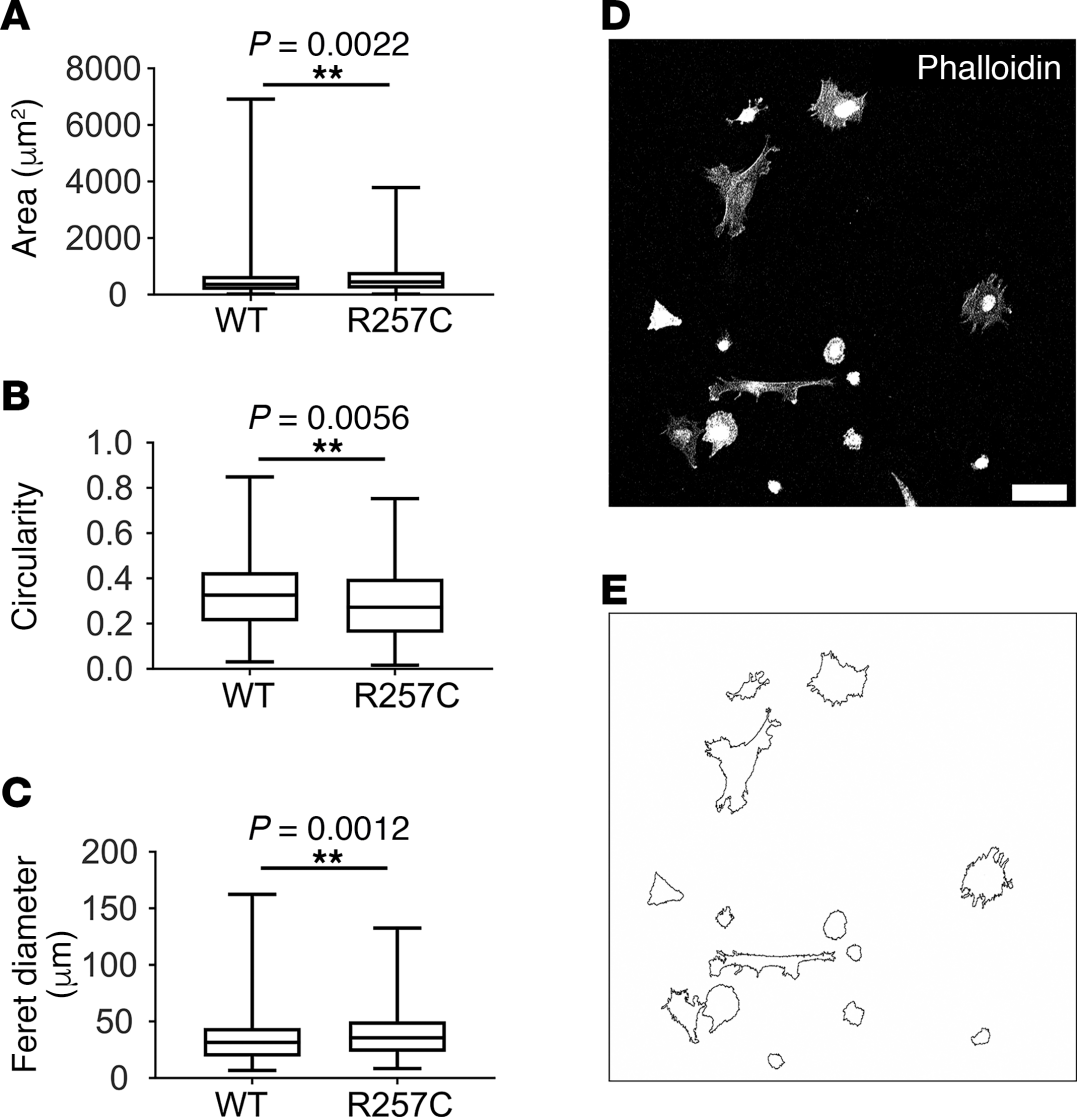
ACTG2^R257C^ enhances HISMC initial spreading. Cell spreading assay showed that 2 hours after seeding, *ACTG2^R257C^*-expressing HISMCs had (**A**) larger area (median [interquartile range], *ACTG2^WT^*, 360.2 μm^2^ (494.6 μm^2^); *ACTG2^R257C^*, 453.2 μm^2^ (588.1 μm^2^); Mann-Whitney test, *P* = 0.0022), (**B**) lower circularity (median [interquartile range], *ACTG2^WT^*, 0.326 [0.2175]; *ACTG2^R257C^*, 0.2725 [0.239]; Mann-Whitney test, *P* = 0.0056), and (**C**) higher Feret diameter (median [interquartile range], *ACTG2^WT^*, 31.56 μm (25.11 μm); *ACTG2^R257C^*, 35.57 μm (26.83 μm); Mann-Whitney test, *P* = 0.0012) compared with those of ACTG2^WT^-expressing HISMCs. (**D**) Representative image of phalloidin-stained HISMCs 2 hours after seeding (scale bar: 50 μm). (**E**) Masks showing outlines of HISMCs in **D**. *n* = 3 independent experiments; ACTG2^WT^, 338 cells; ACTG2^R257C^, 298 cells. Quantitative data are represented as box-and-whisker plots. The box extends from 25th to 75th percentile, and median is indicated by horizontal line. Whiskers represent maximum and minimum values.

To determine if *ACTG2*^R257C^** alters cell spreading, we plated HISMCs expressing *ACTG2*^WT^** or *ACTG2^R257C^* on glass coverslips and fixed them 2 hours later. Phalloidin-stained HISMCs were imaged by confocal microscopy (Figure 7, D and E). Compared with *ACTG2^WT^-*expressing cells, *ACTG2*^R257C^*-*expressing HISMCs spread over a 21% larger area (Figure 7A), were 20% less circular (Figure 7B), and had an 11% greater Feret diameter (Figure 7C) than *ACTG2*^WT^**-expressing HISMCs. Collectively, these data suggest that ACTG2^R257C^ alters actin cytoskeletal dynamics and affects cell movement.

## Discussion

*ACTG2* mutations are the most common cause of visceral myopathy (2). While disease-causing mutations in all other actin isoforms have been studied extensively, pathogenic mutations in *ACTG2* have only recently been discovered (17). Although biochemical and cellular processes affected by *ACTG2* mutations remain poorly defined, symptoms of *ACTG2*-related MMIHS/CIPO suggest that muscle weakness is the primary problem (5). People with myopathic MMIHS/CIPO have very dilated bowels, with minimal contractile force measured by manometry (5). Since force depends on F-actin networks and *ACTG2* encodes the primary actin isoform in visceral SMCs, we used HISMCs that normally produce ACTG2 to test the hypothesis that *ACTG2^R257C^* disrupts actin network architecture or function. Using human visceral SMCs is important because actin is regulated by many proteins and posttranslational modifications, meaning that many aspects of actin biology differ among cell types. To facilitate imaging of actin filament bundles and our rigorous analytical strategy, we cultured HISMCs on glass, recognizing that SMCs in vitro and in vivo have remarkable plasticity and transition to a “synthetic” noncontractile phenotype rapidly in traditional 2D cultures (29). While this phenotypic transition limited some studies, we identified many *ACTG2^R257C^*-induced defects.

Our molecular modeling suggested only minor effects of ACTG2^R257C^ on secondary structure in G-actin or FH2-bound actin monomers. However, R257 is thought to be important for key interstrand contacts needed for actin polymerization and filament bundling (22), and we did not model these interactions or interactions of F-actin with proteins such as tropomyosin. In addition, future modeling might benefit from molecular dynamics simulations. Nonetheless, our in vitro studies support the hypothesis that ACTG2^R257C^ is not efficiently incorporated into actin filaments and that ACTG2^R257C^-containing filaments are less stable than normal.

Quantitative analysis of confocal images showed major deficits in V5-ACTG2^R257C^–containing F-actin bundles compared with V5-ACTG2^WT^ F-actin bundles. Unfortunately, we cannot say if individual ACTG2^R257C^-containing actin filaments are shorter than normal because we only see bundled filaments by light microscopy. However, smaller, shorter, and less elaborately branched F-actin bundles induced by ACTG2^R257C^ could explain the phenotype in MMIHS/CIPO. Specifically, dramatic reductions in V5-ACTG2–containing actin filament bundles relative to V5-ACTG2^WT^ should provide less of a scaffold for myosin-dependent force generation or for latching to prevent passive stretch.

Total V5-ACTG2^R257C^ protein abundance was 43% lower than that of V5-ACTG2^WT^ in transfected HISMCs, even though mRNA levels were similar. This suggests that ACTG2^R257C^ affects protein stability or translational efficiency. Alternatively, increased ACTG2^R257C^ degradation might result from reduced mutant ACTG2 incorporation into actin filaments, as the G-actin/F-actin ratio was 41% higher for V5-ACTG2^R257C^ compared with V5-ACTG2^WT^.

One of our most interesting findings was that deficits seen with V5 antibody were not manifested with phalloidin staining of the exact same *V5-ACTG2^R257C^–*expressing HISMCs. As phalloidin stains all F-actin, absence of quantitative anatomic defects suggests other actin isoforms more robustly incorporate into actin filaments to maintain normal total phalloidin-bound F-actin abundance and structural features in *V5-ACTG2^R257C^–*expressing HISMCs. We confirmed this in several ways. HISMCs expressing untagged *ACTG2^WT^* or *ACTG2^R257C^* had equivalent phalloidin staining. Western blot showed similar actin distributions between Triton X-100–soluble (not cytoskeleton associated) and –insoluble (cytoskeleton associated) fractions using a pan-actin antibody. Electron microscopy, which shows all filaments, confirmed normal appearing F-actin in lamellipodia, filopodia, and stress fibers. Collectively, these results confirm that overall F-actin content and organization was not changed in identifiable ways between *ACTG2^WT^*- and *ACTG2^R257C^*-expressing HISMCs, even though V5-ACTG2^R257C^–containing F-actin was abnormal and mutant protein was more likely to be monomeric than ACTG2^WT^.

One possible explanation for our findings is that ACTG2 forms F-actin networks that are distinct from the total F-actin pool. Consistent with this idea, one study using isoform-specific antibodies showed that ACTG2 was restricted to the central part of cultured porcine vascular SMCs and excluded from contractile fibers penetrating lamella (13). We did not, however, see any difference in distribution for V5-ACTG2^WT^, V5-ACTG2^R257C^, or phalloidin-stained F-actin in various cell regions of HISMCs. We do not know why our results differ from those of Arnoldi (13). It is possible that tagged V5-ACTG2 is not restricted to the center of cells, as is native ACTG2, or that porcine vascular SMCs differ in organization from cultured human bowel SMCs. We note, however, that tagged ACTG2^WT^ and mutant ACTG2 closely associate in actin filament bundles (based on combined V5/FLAG staining), so our results are not explained by restricted localization of the mutant ACTG2 protein.

To determine if ACTG2^R257C^ alters cell biology, we analyzed several F-actin–dependent HISMC functions. Although weak smooth muscle is the hallmark of visceral myopathy, *ACTG2^WT^*- and *ACTG2^R257C^*-expressing HISMCs radially contracted collagen gels equivalently. This suggests sufficiently robust contractile filament bundles in *ACTG2^R257C^*-expressing HISMCs for myosin-induced force generation, at least when SMCs are not well organized. The radial gel contraction assay, however, may not be ideal for assessing contractility, as SMC contractile gene expression depends on spindle cell morphology and parallel cell arrays (30), which are not found in this standard assay. Furthermore, *MYH11* and *ACTA2* mRNA were low in cultured HISMCs we incorporated into collagen gels. In addition, this assay does not measure force generation under stress or ability to passively resist stretch, plausible roles for ACTG2. Consistent with our data, however, vascular SMC contraction was not impaired by ACTG2 knockdown, but was reduced by ACTA2 knockdown (13), an observation that led to the hypothesis that ACTG2 primarily functions in the highly dynamic submembranous actin network (15, 31) instead of in contractile filaments. We therefore evaluated HISMC functions that may depend on this submembranous actin network: cell spreading and cell migration. Remarkably, ACTG2^R257C^-expressing HISMCs spread 21% more and migrated 11% faster than ACTG2^WT^-expressing cells. This is consistent with the observation that contractile cells have thick stress fibers, while migratory cells have less robust stress fibers. Furthermore, moderate myosin inhibition makes fibroblasts migrate faster (21). This may occur because too much contraction makes cell adhesion stronger, which can hinder spreading and migration, processes that require a balance between protrusive and contractile forces (20). Our data therefore suggest that *ACTG2^R257C^* impairs formation and function of contractile stress fibers, resulting in more migratory cells.

There are several additional interesting observations. First, *ACTG2* overexpression dramatically reduced endogenous *ACTG2* mRNA abundance in HISMCs but did not alter abundance of *ACTA2* and *MYH11* mRNAs or the MRTF-A nuclear-to-cytoplasmic ratio protein ratio. This suggests that unlike other SMC contractile genes, *ACTG2* expression might not be MRTF-A–dependent in this system. Our ACTG2^R257C^ data also differ strikingly from other disease-causing ACTG2 mutations studied (17–19). For example, ACTG2 R148S appears to aggregate within SMC in vivo, but we did not see increased actin aggregation in *ACTG2^R257C^*-expressing HISMCs. *ACTG2^R148S^* expression also markedly reduced phalloidin-stained F-actin (although quantitative data were not provided), and ACTG2^R148S^ reduced collagen gel contraction compared with *ACTG2*^WT^-expressing U2OS sarcoma cells. Similarly, *ACTG2^R178L^*, *ACTG2^R178C^*, *ACTG2^R178H^*, *ACTG2^R40C^*, and *ACTG2^R63Q^* expressed in COS-7 or U2OS cells appeared to be inefficiently incorporated into F-actin, and their expression reduced collagen gel contraction (18, 19). Furthermore, Myc-tagged versions of many of these ACTG2 mutants were restricted to the center of the cell, as Arnoldi reported for ACTG2^WT^ (13). However, WT Myc-tagged ACTG2 appeared to extensively colocalize with phalloidin, including at the cell edges, similar to our data with V5-tagged ACTG2^WT^ and mutant ACTG2. While these differences may occur because we studied different ACTG2 mutations, primary human bowel smooth muscle might alternatively possess specialized regulatory mechanisms absent from U2OS sarcoma that maintain normal total F-actin levels when ACTG2^R257C^ is present and that influence ACTG2 localization in filaments. In either case, our data suggest that ACTG2^R257C^ might not be detectable by staining human bowel with phalloidin or with actin antibodies, in contrast to ACTG2^R148S^, which aggregates in SMCs (17).

We also note that biochemical analysis of *ACTA2* with an R258C mutation (same position as R257C in ACTG2) demonstrated altered interactions of actin filaments with myosin and tropomyosin (22). In fact, ACTA2^R258C^-myosin interaction was almost completely abolished if R258C F-actin was bound to tropomyosin. While it is tempting to speculate that mutations in the same in location in ACTA2 and ACTG2 would have similar biological effects, ACTA2 and ACTG2 have been hypothesized to have different roles in SMCs and might be affected differently by a mutation in this position (13, 31). The best evidence of this is that the ACTG2^R257C^ mutation does not cause vascular disease, even though ACTG2 is present in vascular smooth muscle (4, 5, 14, 22, 31). Similarly, ACTA2^R258C^ is not reported to cause visceral smooth muscle disease, even though gut smooth muscle expresses ACTA2. Thus, it is critical that we perform robust, quantitative cellular and biochemical characterization of ACTG2 mutations in relevant cell types to better understand disease pathophysiology.

Extensive quantitative analyses give us high confidence in our results and made it possible to detect problems that were not obvious but raise intriguing questions about ACTG2 function in distinct cell types. There is clearly much more to learn about ACTG2 biology. In the future, we need to study *ACTG2* mutations in contractile visceral SMCs that more closely mimic human bowel in vivo. This could be done using human-induced pluripotent stem cells differentiated into visceral SMCs, but differentiation protocols specific for visceral SMCs are not established. New cell culture models that recreate the normal 3D environment of HISMCs and stretch cells, might also yield functional differences masked by standard tissue culture and randomly aligned cells in radial gel contraction assays. However, the highly detailed quantitative analysis of ACTG2-containing filaments that we performed benefitted from excellent cytoskeleton imaging in SMCs cultured on glass. For example, spindle-shaped cells with very tightly packed actin bundles are challenging to segment for quantitative image analysis. Finally, we need animal models to study the effects of *ACTG2* mutations on whole-organ physiology and survival.

## Methods

### Cell culture.

HISMCs (Sciencell Research Laboratories) derived from human small intestine were cryopreserved after 1 passage. Cryopreserved HISMCs were cultured 1–2 additional passages on tissue culture dishes coated with 0.1% gelatin (MilliporeSigma, G1890) or Matrigel (Corning, 354277) diluted 1:30 in DMEM/F12. All experiments used HISMCs at passage 3–5 in SMC medium (SMCM) (Sciencell, 1101). All incubations were at 37^o^C, 5% CO_2_, in a humidified incubator.

### Plasmids.

*ACTG2*^WT^ and *ACTG2* arginine 257 to cysteine (*ACTG2^R257C^*) mutant cDNA were expressed via constitutive CMV promoter (Supplemental Figure 10, Supplemental Table 1, and Supplemental Methods).

### Overexpression of ACTG2.

Cryopreserved HISMCs plated on 0.1% gelatin-coated tissue culture dishes were allowed to adhere to dishes overnight. Cells at approximately 70%–90% confluence were transfected with *ACTG2* overexpression plasmids (0.278 μg of plasmid/cm^2^ of dish surface area) using Lipofectamine 3000 (Thermo Fisher Scientific, L3000008) according to the manufacturer’s instructions.

### Fluorescence-activated cell sorting.

Forty-eight hours after transfection, HISMC monolayers expressing nuclear tdRFP or nuclear EGFP were washed (1× sterile Dulbecco’s phosphate buffered saline) and trypsinized (0.25% Trypsin-EDTA, 5 minutes, 37^o^C). Trypsin was neutralized using half as much FBS as volume of trypsin. Cells suspended in Iscove’s Modification of DMEM (Corning,10-016-CM) were pelleted (270 *g*, 3 minutes); resuspended in IMDM supplemented with 10% FBS, 10 μL/mL DNaseI (MilliporeSigma, 260913), and 3 μL/mL monothioglycerol (MilliporeSigma, M6145); and sorted on a BD FACSJazz (BD Biosciences), MoFlo Astrios (Beckman Coulter), or BD FACSAria Fusion (BD Biosciences) to isolate tdRFP^+^ or EGFP^+^ cells. Sorted cells were resuspended in SMCM (Sciencell, 1101) before study.

### RNA isolation and purification.

RNA was purified from cells lysed in TRIzol (Ambion, 15596018) using the RNeasy Plus Mini kit (QIAGEN, 74134), with RNase Free DNase Set (QIAGEN, 79254) to remove residual DNA. RNA concentrations were measured by NanoDrop (ND-2000, Thermo Fisher Scientific). To isolate RNA from freshly resected human colon smooth muscle, approximately 5 mm longitudinal smooth muscle pieces were pulled off the tissue using sharp forceps. The tissue was minced using sharp scissors followed by addition of 1 mL TRizol per 50 mg of tissue and vortexing for 15 minutes. The samples were subsequently spun down at 1200 *g* for 3 minutes to pellet tissue debris, and the supernatant was transferred to a fresh tube. RNA was purified as above for cells.

### Quantitative real-time PCR.

Quantitative real-time PCR (qRT-PCR) was performed using SsoFast Evagreen Supermix with Low ROX (Bio-Rad, 172-684 5211) and previously described primers (where possible) or new primers validated using freshly isolated human gut smooth muscle (Supplemental Table 2). PCR product size was confirmed by agarose gel electrophoresis. Cycle threshold (C_t_) values were normalized to *YWHAZ* mRNA.

### Quantification of endogenous versus exogenous (overexpressed) ACTG2.

We quantified the relative expression of transfected *ACTG2^WT^* or *ACTG2^R257C^* compared with endogenous *ACTG2* using a common forward primer (*ACTG2 3*′ Endo/Exo forward) and unique reverse primers (reverse primer for endogenous *ACTG2* = *ACTG2* 3′ endo reverse; reverse primer for overexpressed *ACTG2* = IRES reverse qPCR). Primer sequences are provided in Supplemental Table 2. We generated standard curves for the 2 different primer pairs and used these standard curves to determine the expression of endogenous and exogenous *ACTG2*.

### Immunofluorescent staining.

HISMCs on glass coverslips were washed with PBS, fixed (4% paraformaldehyde, 30 minutes, room temperature), washed twice with PBS, blocked (5% normal donkey serum [NDS], 0.5% Triton X-100 in PBS [0.5% PBST]) (1 hour, room temperature), incubated in primary antibodies (5% NDS, 0.5% PBST 1 hour, room temperature) (Supplemental Table 3), washed 3 times for 5 minutes (0.5% PBST), and then incubated in secondary antibodies (Supplemental Table 3) (0.5% PBST, 30 minutes, dark, room temperature). Phalloidin staining was performed after secondary antibodies by washing 3 times for 5 minutes (PBS) and incubating (1 hour, dark, room temperature) in Alexa Fluor–conjugated phalloidin (488, 555, or 647; Invitrogen) diluted 1:50 in PBS. Cells were washed 3 times in PBS, incubated in Hoechst 3342 nuclear dye (1:1000, Invitrogen, H3570) (30 minutes, room temperature), washed twice in PBS, mounted in Prolong Gold AntiFade Mountant (Life Technologies, P36934), and allowed to set (overnight, dark, room temperature) before long-term storage at 4°C.

### Immunofluorescence microscopy.

Coverslips were imaged using a Zeiss LSM 710 laser scanning confocal microscope and Zeiss ZEN 2.3 SP1 FP3 (black) (version 14.0.18.201) software. Images were acquired with a ×20/0.8 air or ×63/1.4 oil DIC M27 Plan-Apochromat objective. The Zeiss LSM 710 condenser numerical aperture is 0.55. Confocal images were processed in ImageJ (NIH) to crop, scale, and uniformly color adjust. Confocal images are represented as “sum of slices” or “maximum intensity” projections after ImageJ processing.

### Quantitative image analysis.

Imaris (version 9.02, Bitplane Inc.) was used to characterize actin filament bundle structures in confocal *Z*-stack images of HISMCs expressing V5-*ACTG2*^WT^** or *ACTG2^R257C^*. Cells expressing V5-tagged *ACTG2* were manually segmented based on V5 signal to generate isosurfaces. Cell volume, sphericity, total V5, and total phalloidin intensity were obtained from the isosurfaces. Local contrast (defined by approximate filament bundle diameter) was used to segment isosurfaces corresponding to filament bundles based on V5 and phalloidin staining. Total V5 intensity in filaments was obtained for isosurfaces of filament bundles. Total V5 intensity in filaments is normalized to total V5 intensity in cells. Isosurfaces of filament bundles were used to generate 3D network reconstructions of filament bundles. Parameters calculated for filament bundles: total volume/cell, total length/cell, average diameter/cell, average length of longest projection in 3D space, total number of branch points/cell, and average straightness/cell. Identical parameters were used to analyze *ACTG2^WT^*- and *ACTG2^R257C^*-expressing HISMCs (Supplemental Methods and Supplemental Data 1).

### Detergent extraction of cytoskeleton.

HISMCs 24 hours after transfection with *ACTG2*^WT^*-* or *ACTG2^R257C^*-IRES-ntdRFP were isolated by flow cytometry for tdRFP fluorescence and seeded (50,000 cells/well) on 0.1% gelatin-coated 18 mm glass coverslips in 12-well dishes. Twenty-four hours after seeding, media was aspirated and cells were washed once with PBS with Ca^2+^/Mg^2+^. Extraction solution (Supplemental Methods) was added to each well for 5 minutes. Cells were then rinsed once with PEM buffer and fixed (4% paraformaldehyde, 30 minutes, room temperature). Wells were washed twice with 1× PBS before Alexa Fluor 488 phalloidin (Life Technologies, A12379) staining.

### Triton X-100–insoluble cytoskeleton assay and Western blot.

Ratios of monomeric to filamentous actin were determined by Western blot after fractionating into Triton X-100–soluble and –insoluble fractions (Supplemental Methods). HISMCs expressing V5-*ACTG2*^WT^*–* or *ACTG2^R257C^*-IRES-nEGFP were isolated by flow sorting for EGFP 48 hours after transfection and then grown 24 hours on 0.1% gelatin-coated 6-well dishes. Cells were washed twice (1× PBS). Soluble fraction was collected in 100 μL 1% Triton X-100 lysis buffer/well. Remaining insoluble fraction protein was extracted with 100 μL of 1% SDS lysis buffer/well and collected using a rubber scraper. Fractions were triturated 10 times (P200 pipet) followed by vortexing (30 seconds). After centrifugation (14,000 *g*, 15 minutes, 4^o^C), supernatant was transferred to a fresh tube, mixed with 40 μL 4× loading buffer (Li-Cor, 928-40004) and 16 μL 10× NuPAGE Sample Reducing Agent (Invitrogen, NP0009), vortexed briefly, heated (95°C, 10 minutes), and stored at –20°C. Soluble and insoluble fractions separated by SDS-PAGE were probed using V5 and pan-actin antibodies (Supplemental Table 3) alongside Odyssey One-Color Protein Molecular Weight Marker (Li-Cor, 928-40000). Protein was transferred to nitrocellulose membranes using iBlot transfer machine (Invitrogen, Life Technologies, IB1001). Membranes were imaged using Li-Cor Odyssey 9120 Infrared Imaging System. Ratios of soluble to insoluble protein were calculated by dividing background normalized mean band intensities.

### Functional assays.

*ACTG2*^WT^**- or *ACTG2^R257C^*-IRES-ntdRFP–expressing HISMCs were isolated by flow sorting for tdRFP^+^ cells 24 hours after transfection.

### Collagen gel contraction assay.

Cells were resuspended in SMCM (1.67 × 10^5^ cells/mL). Rat tail collagen-1 (Corning, 354236) in 0.1% acetic acid was added (final concentration 1.5 mg/mL) and triturated with a P1000 pipet. The minimum volume of 1 M sodium hydroxide needed to neutralize acetic acid and induce collagen-1 gel formation was determined empirically. Immediately following addition of collagen-1, sodium hydroxide was added and rapidly mixed with P1000 pipet. 300 μL collagen/HISMC suspension was added to each well of a 48-well tissue culture dish. Gel was allowed to set for 20 minutes at room temperature. 500 μL SMCM was added on top of the gel and a P200 pipet tip was used to release gels from walls of wells. Pictures of gels were taken at 0, 24, 48, and 72 hours using a 13-megapixel dual rear camera (Moto G5S Plus smartphone). Gel contraction was determined by normalizing gel area at each time point to *t* = 0 hours gel area.

### Cell spreading assay.

HISMCs resuspended in SMCM were seeded on 18 mm glass coverslips in 12-well tissue culture dishes (50,000 cells/well). Two hours after seeding, cells were fixed (4% paraformaldehyde, 30 minutes) and stained with Alexa Fluor 647 phalloidin. Confocal images were quantified using ImageJ (NIH). Phalloidin staining was used to create masks of cell surfaces. Shape descriptors (area, circularity, Feret diameter) were measured using the *Measurement* tool in ImageJ.

### Cell migration assay.

Cells resuspended in SMCM were seeded in 24-well tissue culture dishes (30,000 cells/well). Six to eight hours after seeding cells, 100 nM SiR-Actin (CY-SC001) was added to each well for 24 hours to aid visualization. Confocal images taken every 10 minutes for 12 hours were analyzed with Imaris (Bitplane Inc.). The *Spots* tool was used to track nuclear tdRFP (Supplemental Data 2). Mean track speed and persistence were obtained from Imaris data.

### Statistics.

GraphPad Prism (version 7.03) was used for all statistical analyses. Two-tailed Student’s *t* test (parametric data) or Mann-Whitney rank sum test (nonparametric data) was used for comparisons between 2 groups. When multiple groups were compared, 1-way ANOVA was used for parametric data and Kruskal-Wallis tests were used for nonparametric data, with multiple comparison correction. Significance cutoff was set at *P* < 0.05. Data are represented as mean ± SEM for parametric data and median (interquartile range) for nonparametric data. The same imaging and analysis parameters were applied to *ACTG2^WT^* and *ACTG2^R257C^* samples for all analyses.

### Study approval.

Deidentified human colon samples were collected under the protocol approved by the Children’s Hospital of Philadelphia (IRB 13-010357) and the Perelman School of Medicine at the University of Pennsylvania (IRB 804376).

## Author contributions

SKH designed the study, developed the methodology, curated the data, performed formal analysis, wrote the manuscript, and obtained funding. VB and CY assisted in data curation, formal analysis, and writing the manuscript. SS assisted in study design, developing the methodology, and reviewing and editing the manuscript. TMS was involved in data curation, formal analysis, reviewing and editing the manuscript, and providing funding. ROH was involved in study design, reviewing and editing the manuscript, project administration, and providing funding.

## Supplementary Material

Supplemental data

Supplemental Video 1

## Version 1 08/20/2020

Electronic publication
