# Supplementary material for: Pseudo-obstruction–inducing ACTG2R257C alters actin organization and function
Source: JCI Insight. 2020 Aug 20;5(16):e140604. doi: 10.1172/jci.insight.140604 (PMC7455133; doi:10.1172/jci.insight.140604)
Supplement: Supplemental data [file jciinsight-5-140604-s183.pdf]

Supplementary Methods

Three-dimensional modeling of R257C

Creating the Mutant Proteins

PDB structures of mutant ACTG2 were created using Modeller 9.23 (23). Since Modeller is a non-graphical software, comparative modelling was done using UCSF Chimera (32). First, desired sequence (changing arginine 257 to cysteine from the normal sequences 1j6z and 1y64) was aligned with the template sequence (from the respective 1j6z and 1y64) using Tools → Blast Protein → MultAlignViewer. Once the alignment of the structures was achieved, Modeller was used to create five output models (in the MultAlign Viewer window: Structure → Modeller (Homology)). After the output models were produced, the one with the best alignment and lowest energy (lowest DOPE, -0.38 for both) was chosen as structure of the mutant protein. DOPE stands for Discrete Optimized Protein Energy and is a statistical potential or energy function of the protein.

Protein Dynamics Analysis using MolProbity

MolProbity is an online protein modeling tool that computes all-atom contact in proteins (25). The pdb file for each protein was uploaded and hydrogens were added again using electron-cloud x-H bond length (for x-ray crystal structures) since they are removed by the initial processing of the structures. To optimize the energetics of the structure, the suggested residues were also flipped. A series of parameters (clashes, angles, deviations) were obtained through the 'Analyze all-atom contacts and geometry' option. Specific contacts between each residue and every other residue in the protein were probed through the 'Visualize interface contacts' option.

Plasmid Construction

WT *ACTG2* cDNA was PCR amplified from human colon smooth muscle (Supplementary Table 1) and cloned into pEGFP-C1 Lifeact-EGFP (RRID:Addgene_58470) after digestion with NheI and PstI to remove Lifeact-EGFP. Internal ribosome entry site (IRES) and tandem-dimer red

fluorescent protein (tdRFP) were PCR-amplified from a plasmid gifted by Children's Hospital of Philadelphia Human Pluripotent Stem Cell Core. Phusion High-Fidelity DNA Polymerase (New England Biolabs, MA, USA; Catalog#M0530L) was used to amplify all cDNA. Nuclear-localization signal (NLS) was included in forward primer for amplifying tdRFP. Reverse primer included IRES sequence. PCR products were agarose gel purified using NucleoSpin Gel and PCR Clean-Up kit (Machery-Nagel,PA, USA; Cat#740609). pC1-*ACTG2*-IRES-ntdRFP plasmid was constructed using Gibson Assembly Master Mix (New England Biolabs, Catalog#E2611). To construct V5-*ACTG2* and FLAG-*ACTG2* overexpression plasmids, pC1-*ACTG2*-IRES-ntdRFP was digested with AscI/XhoI. V5 (GGTAAGCCTATCCCTAACCCTCTCCTCGGTCTCGATTCTACG) or FLAG (GACTACAAGGACGACGATGACAAG) gBlocks (Integrated DNA Technologies, IA, USA; IDT) were inserted by Gibson Assembly. To create V5/FLAG-*ACTG2* or V5/FLAG-*ACTG2*-IRES-nEGFP, IRES-ntdRFP sequence was removed by restriction digest with AgeI/NheI (for V5) or AgeI/XbaI (for FLAG). After gel purifying, plasmid was resealed with T4 DNA Ligase (New England Biolabs, MA, USA, Catalog#M202) to create V5/FLAG-*ACTG2* overexpression plasmid. To create V5/FLAG-*ACTG2*-IRES-nEGFP, digested *ACTG2*-IRES-ntdRFP backbone was T4 ligated with compatible sticky ends using a gBlock for IRES-NLS-EGFP (Integrated DNA Technologies, IDT). *R257C* mutation was introduced in all plasmids using QuikChange II site-directed mutagenesis with Pfu Ultra-High Fidelity DNA Polymerase (Agilent, CA, USA; Catalog#600380), via primers: Forward 5'→3' CATTGGCAATGAGCGCTTCTGCTGCCCTGAGACCCTCTTC; Reverse 5'→3' GAAGAGGGTCTCAGGGCAGCAGAAGCGCTCATTGCCAATG. All plasmids were verified by Sanger sequencing. Primers are in Supplementary Table 1. Plasmid maps are in Supplementary Figure 10.

Human Intestinal Smooth Muscle Cell Expansion

To expand purchased cells, we plated $5x10^5$ HISMCs on 10 cm tissue culture dishes coated with 2 μg/cm^2 poly-L-lysine (Sciencell, Catalog#0413) in smooth muscle cell medium (SMCM) (Sciencell, Catalog#1101) with 2% fetal bovine serum (FBS) (Sciencell, Catalog#0010), 1% Penicillin/Streptomycin (Sciencell, Catalog#0503), and smooth muscle cell growth supplement (Sciencell, Catalog#1152). Media was changed every other day. When HISMCs were confluent we cryopreserved in 90% FBS/10% dimethyl sulfoxide (DMSO) at $5x10^5$ cells/mL.

Quantitative Image Analysis in Imaris

Imaris (Version 9.2, Bitplane Inc.) was used to characterize actin filament bundle structure in 0.3 μm confocal Z-stack 20x images of HISMCs expressing V5-*ACTG2 WT* or V5-*ACTG2-R257C*. Cells expressing V5-tagged *ACTG2* were manually segmented based on V5 signal to generate isosurfaces. Total V5 and phalloidin intensity were obtained from *Intensity Sum* parameter. Using *Background subtraction*, local contrast (defined by approximate filament bundle diameter) was used to segment isosurfaces corresponding to filament bundles based on V5 and Phalloidin staining. Total V5 intensity in filaments was obtained from *Intensity Sum* parameter for isosurfaces corresponding to filament bundles. Total V5 intensity in filaments is normalized to total V5 intensity in cells. Isosurfaces corresponding to filament bundles were used to generate 3D reconstruction of filament bundles using *Filament Tracer* tool. Parameters calculated for filament bundles: total volume/cell, total length/cell, average diameter/cell, average length of longest projection in 3D space (*BoundingBoxOO Length*), total number of branch points/cell, and average straightness/cell. Identical parameters were used to analyze *WT* and *R257C*-expressing HISMCs (see Supplementary Data; The .icpx files can be directly imported into Imaris). For MRTF-A nuclear:cytoplasmic (N:C) ratio, Hoechst staining was used to segment the nucleus. MRTF-A staining in cytoplasm was obtained by subtracting MRTF-A pixel intensity in nucleus

from total MRTF-A pixel intensity through the z-stack using cell isosurfaces previously generated based on V5 signal. Nuclear intensity was divided by cytoplasmic intensity to calculate N:C ratio.

Extraction buffer

0.5% Triton X-100 and 0.25% Glutaraldehyde in PEM buffer (100 mM PIPES-KOH (pH6.9), 1 mM EGTA, 1 mM $MgCl_2$.

***ACTG2* WT and *ACTG2* R257C Co-transfection**

HISMCs were transfected with *FLAG-ACTG2 WT-IRES-ntdRFP* plus *V5-ACTG2 R257C-IRES-nEGFP* or *FLAG-ACTG2 R257C-IRES-ntdRFP* plus *V5-ACTG2 WT-IRES-nEGFP* keeping total DNA constant (0.278 µg of plasmid/cm^2). After 48 hours, HISMCs sorted for tdRFP/EGFP double positive cells, were seeded on glass coverslips coated with 0.1% gelatin, cultured 24 hours, and fixed (4% paraformaldehyde, 30 minutes). HISMCs stained with V5 (species: rabbit) and FLAG (species: mouse) antibodies (Supplementary Table 3), followed by secondary antibodies – Alexa Fluor Donkey anti-Rabbit 555 and Alexa Fluor Donkey anti-Mouse 488 were imaged (ZEISS LSM 710 Laser Scanning Confocal Microscope, 63X oil objective) to visualize filament bundles in defined cell regions. Images were processed (ImageJ) to generate projections of confocal Z-stacks using ‘Sum of Slices’.

Platinum Replica Electron Microscopy (PREM)

HISMCs 48 hours post-transfection with ACTG2 WT-IRES-ntdRFP or ACTG2 R257C-IRES-ntdRFP were sorted to isolate tdRFP+ cells, seeded on gelatin-coated glass coverslips, and cultured overnight and then extracted using 1% Triton X-100 in PEM buffer (100 mM PIPES-KOH, pH 6.9, 1 mM MgCl2, and 1 mM EGTA) containing 2% polyethylene glycol (PEG) (MW 35,000), 2 µM phalloidin and 10 µM taxol for 3 min at room temperature. After three quick rinses with PEM buffer containing 2 µM phalloidin and 10 µM Taxol, extracted cells were fixed (2% glutaraldehyde

in 0.1 M sodium cacodylate buffer (pH 7.3), 20 min) (33) and processed for PREM as previously described (28). Briefly, glutaraldehyde-fixed cells were post-fixed sequentially with 0.1% tannic acid and 0.2% uranyl acetate in water, dehydrated in graded ethanol series (10%, 20%, 40%, 60%, 80% and twice 100% ethanol for 5 min each), treated with 0.2% uranyl acetate in 100% ethanol (20 min), and washed (100% ethanol four times for 5 min). Samples were then critical-point dried and coated with platinum and carbon before imaging using JEM 1011 transmission electron microscope (JEOL USA, Peabody, MA) operated at 100 kV. Images were acquired via ORIUS 810 W CCD camera (Gatan, Pleasanton, CA) and presented in inverted contrast.

Triton-insoluble cytoskeleton assay buffers

1% Triton X-100 lysis buffer: 1% Triton X-100, 50 mM NaCl, 1 mM $MgCl_2$, 10 mM HEPES (pH 7.0), 2.5 mM EGTA, and 1x Complete Mini Protease Inhibitor Cocktail (Roche; Catalog#4693124001)

1% SDS lysis buffer: 1% SDS (10% SDS solution, Invitrogen; Catalog#15553027), 50 mM NaCl, 1 mM $MgCl_2$, 10 mM HEPES (pH 7.0), 2.5 mM EGTA, and 1x Complete Mini Protease Inhibitor Cocktail (Roche; Catalog#4693124001)

Supplementary Figure 1. Three-dimensional modeling of ACTG2 R257C

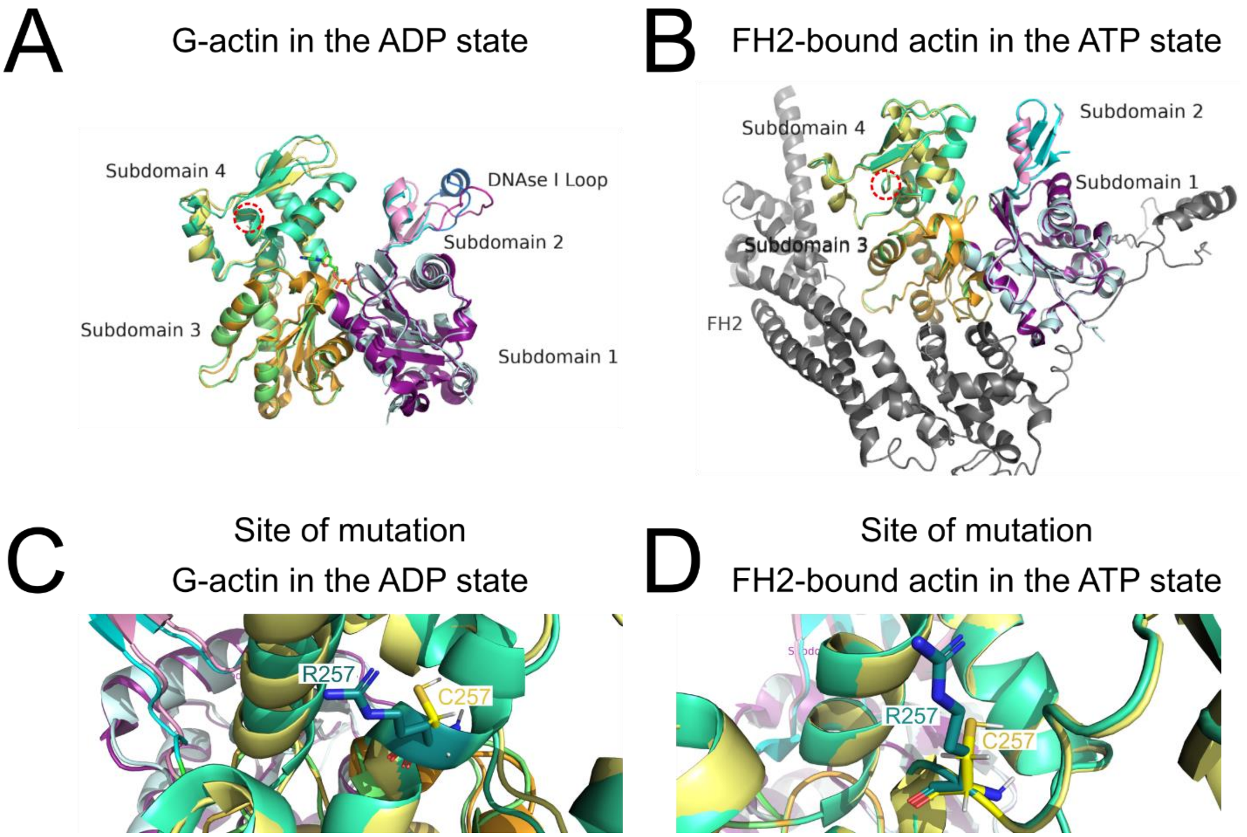

The four actin subdomains are illustrated: subdomain 1 (WT: dark-purple, R257C: light-cyan); subdomain 2 (WT: dark-cyan, R257C: light-pink), which in G-actin also includes a DNAse I Binding Loop (WT: dark-blue, R257C: dark-pink); subdomain 3 (WT: yellow-orange, R257C: light-green); subdomain 4 (WT: green-cyan, R257C: light-yellow). A) G-actin in the ADP state (1j6z) with WT and corresponding Modeller-generated R257C mutant superimposed. B) FH2-bound actin in the ATP state (1y64) as a proxy for F-actin and the corresponding Modeller-generated R257C mutant. C) Site of mutation (red dotted circle in A) in G-actin in the ADP state (shown as R257) and the Modeller-generated mutant (C257). D) Site of mutation (red dotted circle in B) in FH2-bound actin in the ATP state (shown as R257) and the Modeller-generated mutant (C257).

Supplementary Figure 2. Ramachandran plots

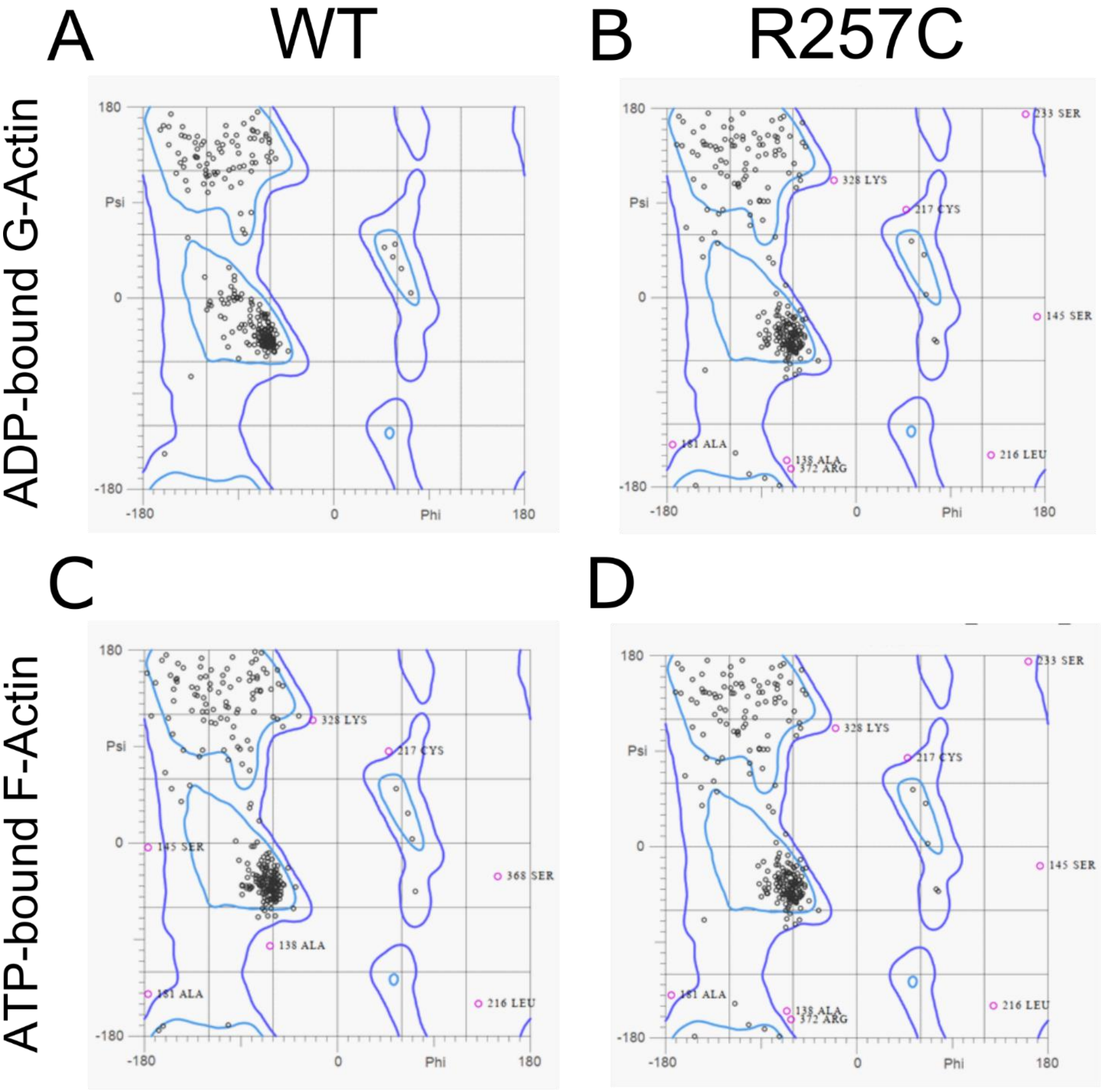

Ramachandran plots of WT G-actin (A), R257C G-actin (B), WT F-actin (C), R257C F-actin (D). Dark blue contour highlights the allowed dihedral angles. Light blue contour highlights the favorable dihedral angles. The black circles represent all residues. Pink circles represent residues that are outliers.

Supplementary Figure 3. Potential interaction of R257C with C218

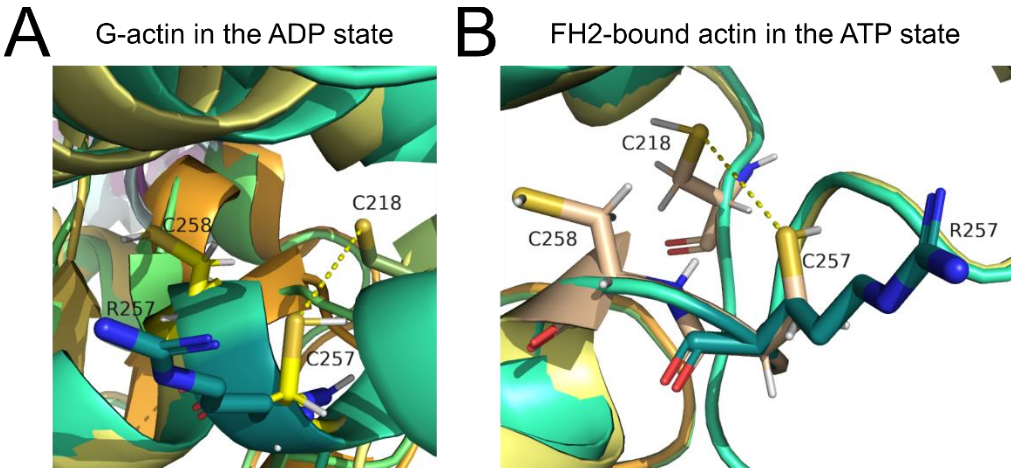

Potential interaction of the mutated amino acid (C257) with C218. R257 of the normal G-actin protein is also shown to illuminate the differences in angles and potential interactions between WT and mutant protein at this site. While the distance might not favor a disulfide bond, these structures were generated using Modeller which does not take into consideration the thermodynamics involved in the formation of a disulfide bond (Distance between Cysteines in ADP-bound G-actin: 6.3 Å; Distance between Cysteines in ATP-bound F-actin: 6.7 Å; Disulfide bond length is about 2.05 Å) (26).

Supplementary Figure 4. Most experiments were performed with flow sorted HISMCs transfected with *ACTG2 WT-IRES-ntdRFP* or *ACTG2 R257C-IRES-ntdRFP*.

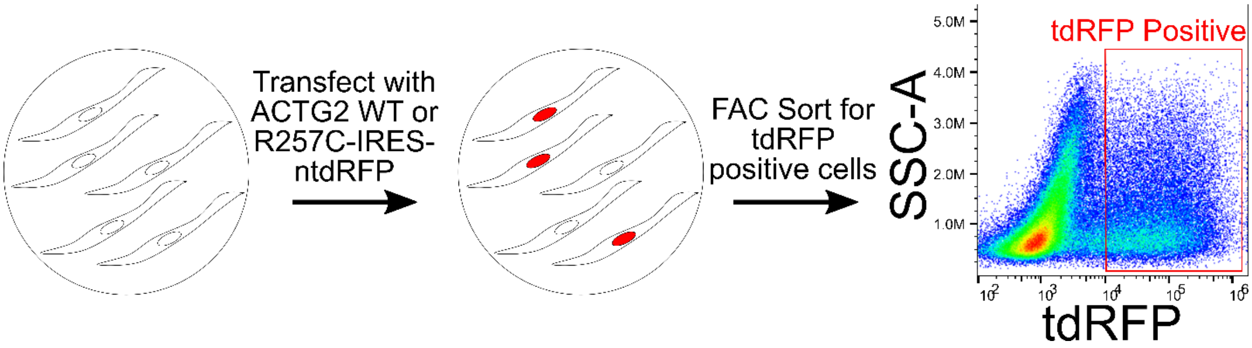

Some experiments were performed with V5-ACTG2-IRES-ntdRFP, V5-ACTG2-IRES-nEGFP, or FLAG-ACTG2-IRES-ntdRFP, when tagged ACTG2 was required in addition to sorted cells.

Supplementary Figure 5. MRTF-A Nuclear to Cytoplasmic ratio in HISMCs expressing V5-ACTG2 WT or V5-ACTG2 R257C

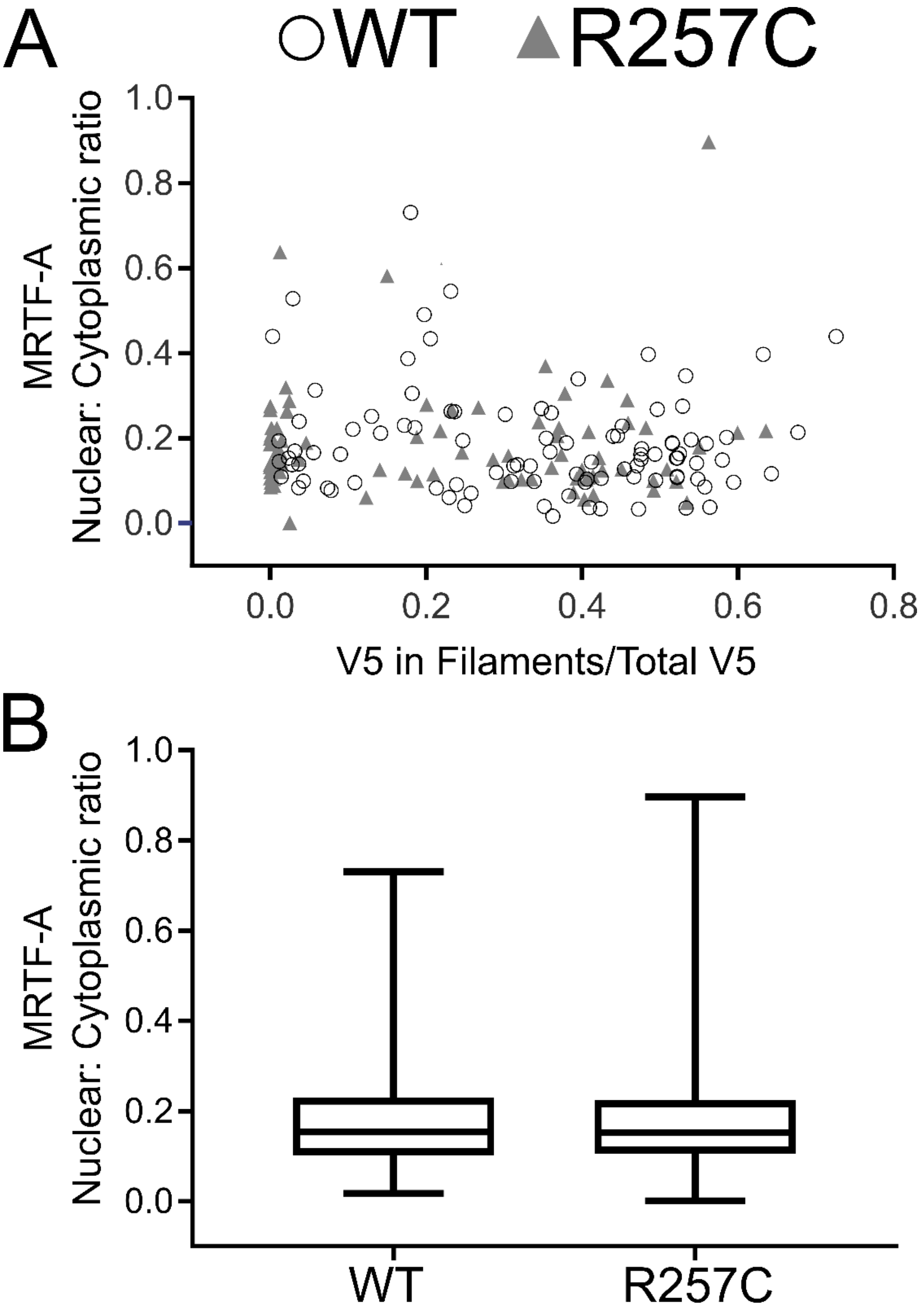

MRTF-A nuclear to cytoplasmic ratio has a similar distribution in HISMCs expressing V5-ACTG2 WT or R257C A) relative to the proportion of V5 in filaments and B) absolute MRTF-A Nuclear: Cytoplasmic ratio (N=3; WT 97 cells, R257C 84 cells, Mann-Whitney *P=.9130*)

Supplementary Figure 6. Filament bundle straightness is unaffected by ACTG2 R257C

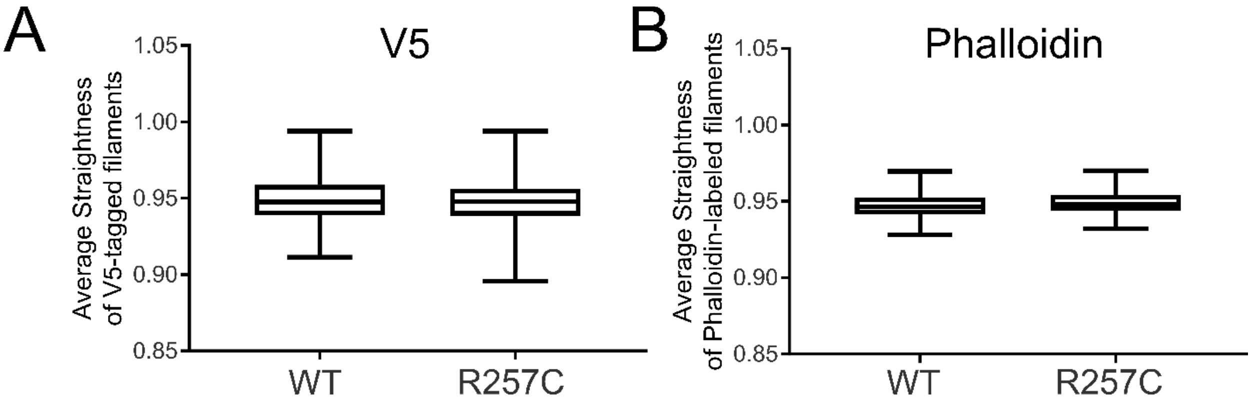

There is no difference in average straightness (length of filament/shortest distance between the start and end of filament) for either A) the V5-tagged filaments or B) the Phalloidin-labeled filaments.

Supplementary Figure 7. V5-ACTG2 WT or R257C have equivalent overlap with phalloidin in central and peripheral cell regions (Scale bar 20 μm).

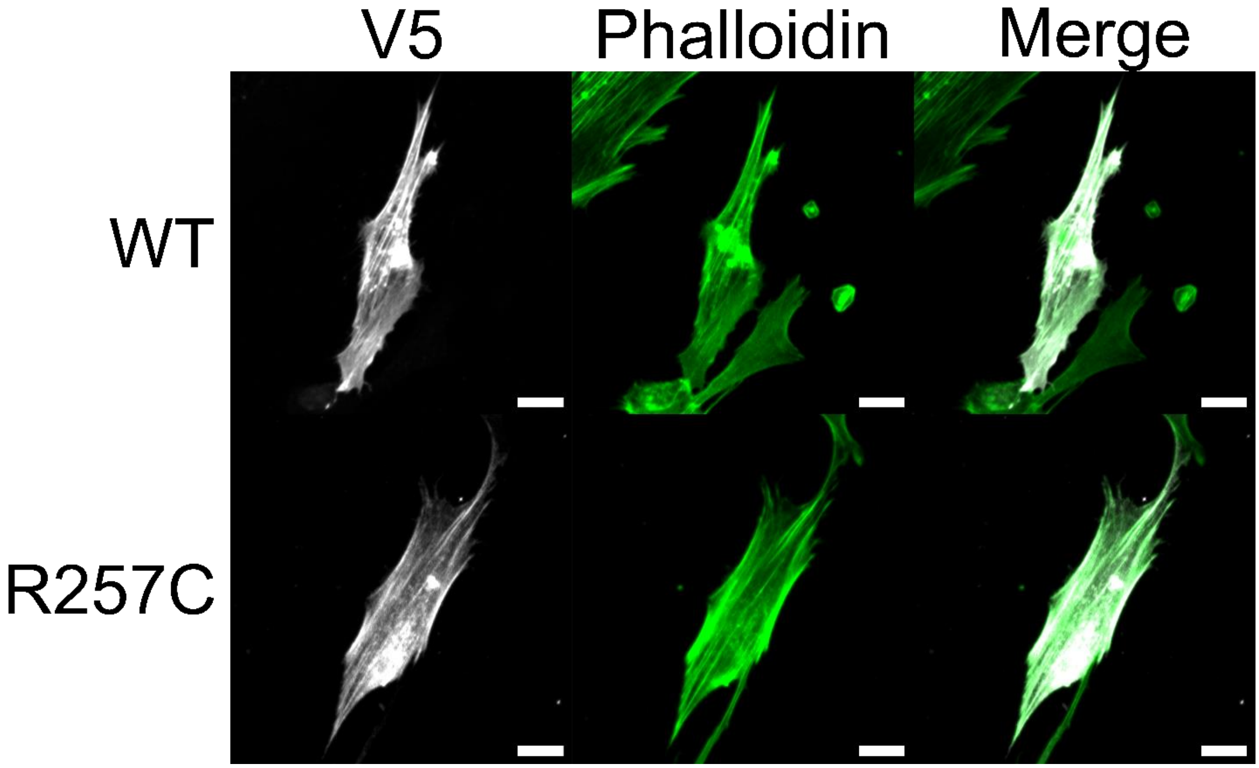

Supplementary Figure 8. Co-transfected ACTG2 WT and R257C shows excellent overlap.

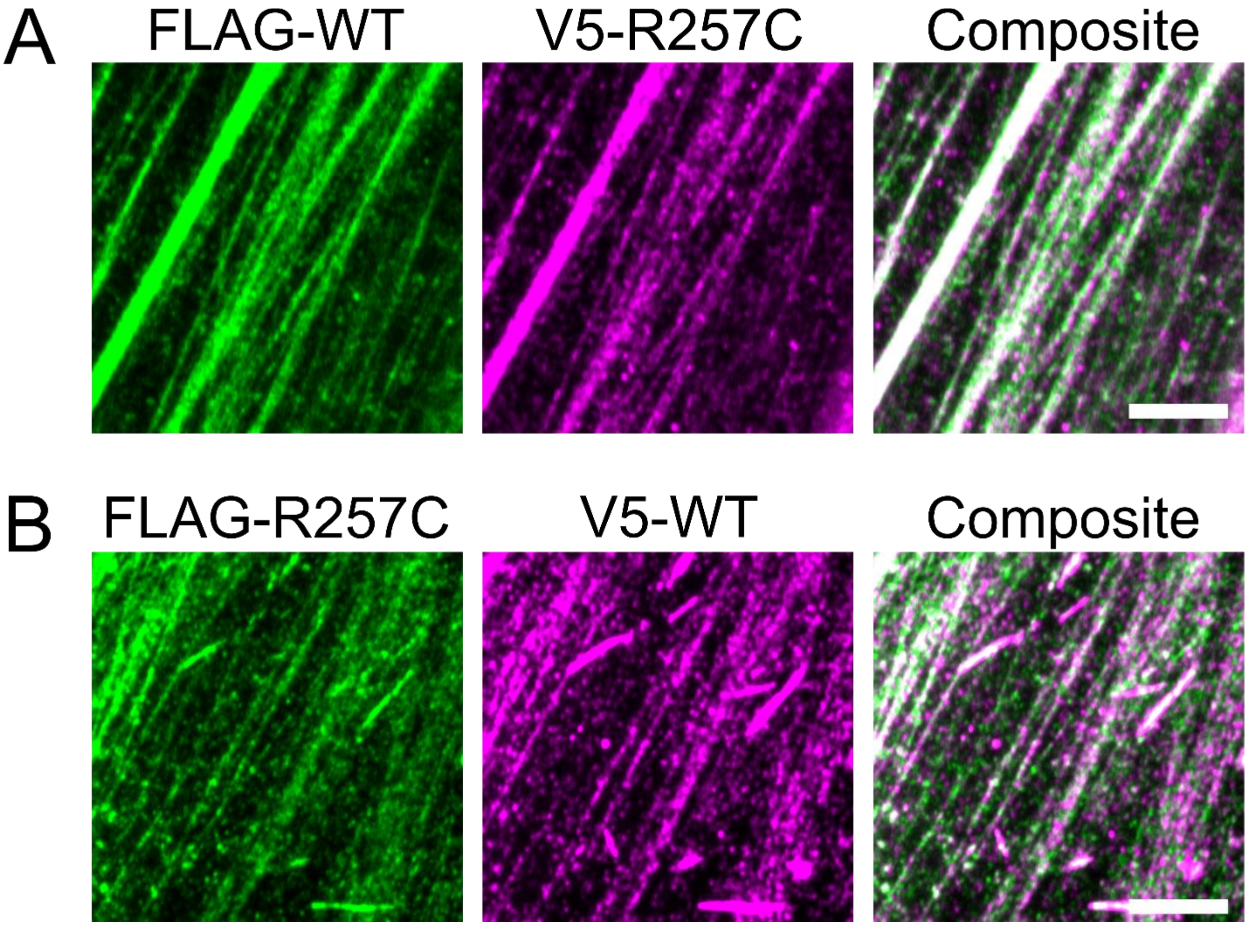

63x oil objective confocal Z-stacks (Sum of Slices) of HISMCs co-transfected with A) *FLAG-ACTG2 WT-IRES-ntdRFP* and *V5-ACTG2 R257C-IRES-nEGFP*, or B) *FLAG-ACTG2 R257C-IRES-ntdRFP* and *V5-ACTG2 WT-IRES-nEGFP* (Scale bar 5 μm). Co-transfected tdRFP/EGFP double positive HISMCs isolated by flow cytometry were stained for V5 (Magenta) and FLAG (Green). There is excellent overlap between ACTG2 WT and ACTG2 R257C in filament bundles.

Supplementary Figure 9. ACTG2 R257C does not cause obvious ultrastructural defects in F-actin networks.

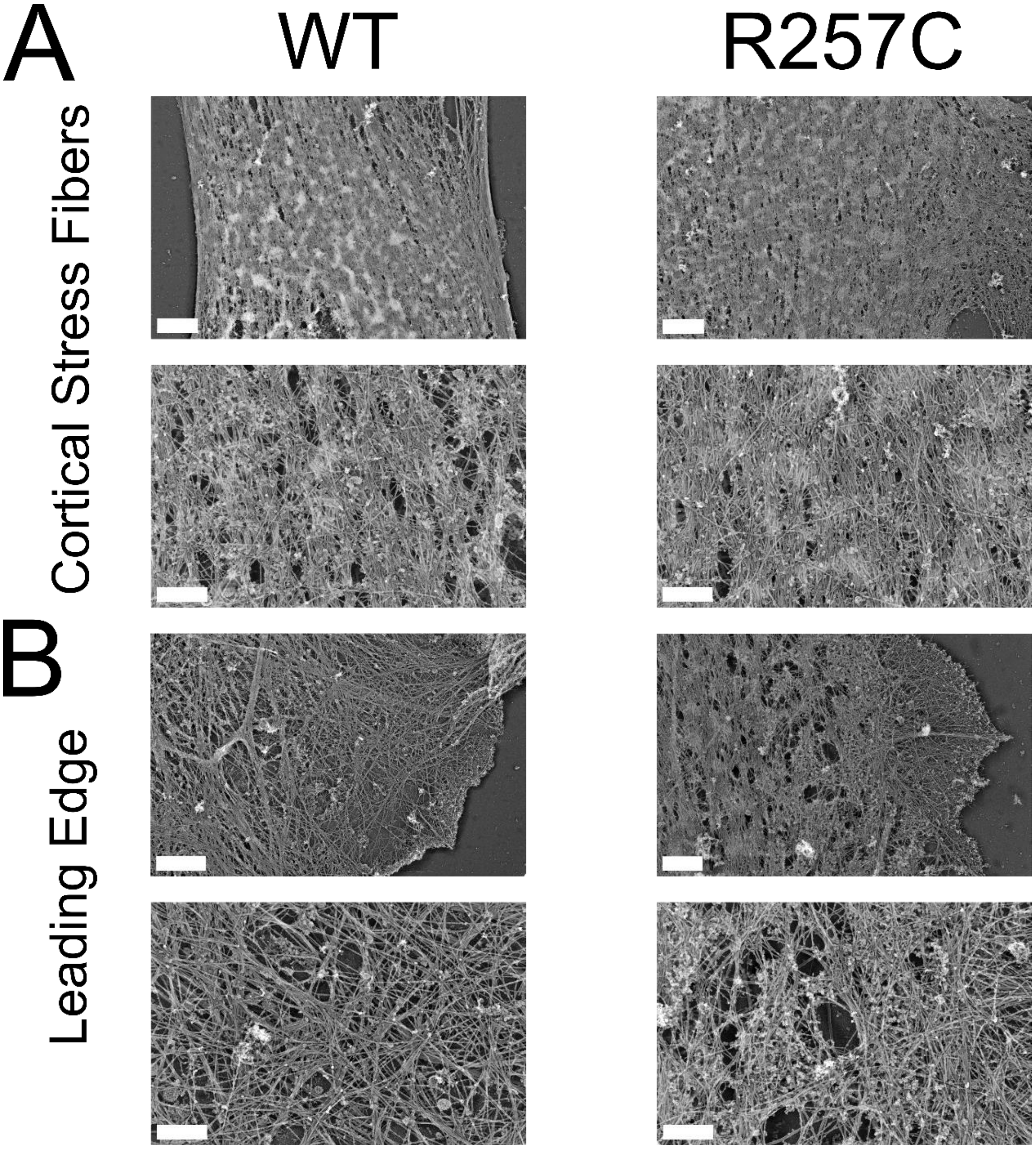

Platinum replica electron microscopy (PREM) of sorted HISMCs expressing ACTG2 WT-IRES-ntdRFP (n=16 cells) or ACTG2 R257C-IRES-ntdRFP (n=22 cells) showed similar F-actin networks. A) Cortical stress fibers between the nucleus and the leading edge of the cell. Upper left: WT (Scale bar 2 μm). Lower left: Zoomed in image of cortical stress fibers in WT-expressing HISMCs (Scale bar 500 nm). Upper right: R257C (Scale bar 2 μm). Lower right: Zoomed in image of cortical stress fibers in *R257C*-expressing HISMCs (Scale bar 500 nm). B) Actin filaments in lamella and lamellipodia at cell leading edge. Upper left: WT (Scale bar 2 μm). Lower left: Zoomed in image of actin filaments in cell leading edge of WT-expressing HISMCs (Scale bar 500 nm). Upper right: R257C (Scale bar 1 μm). Lower right: Zoomed in image of actin filaments in the leading edge of R257C-expressing HISMCs (Scale bar 500 nm).

Supplementary Figure 10. Plasmid Maps

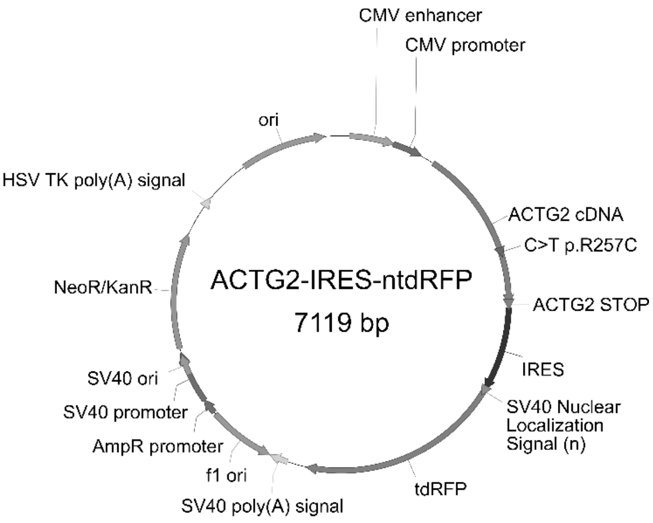

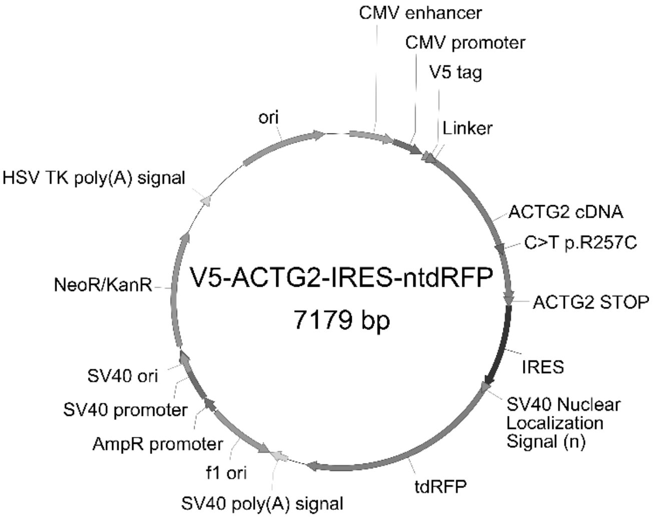

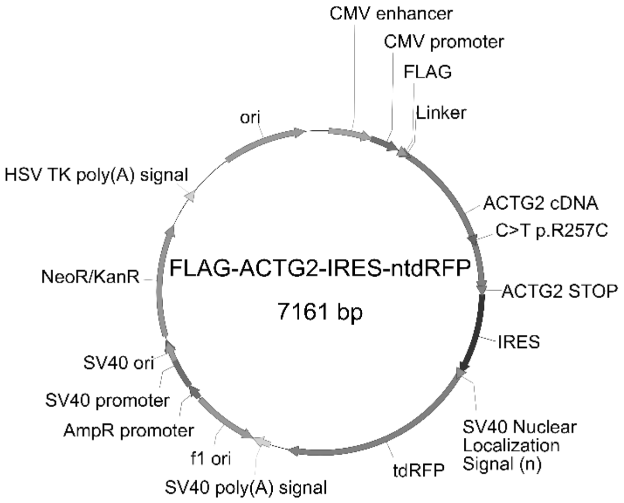

Supplementary Table 1. Overexpression vector design PCR primers

**Primer Name**	**Sequence 5’ → 3’**	**T_m (ºC)**	**GC (%)**
ACTG2 5'UTR F1	GTTGTTGACAGCTTCAAGTCG	56	48
ACTG2 3'UTR R1	CACCTACCTTACAGCACTATTGC	57	48
ACTG2 cDNA Fwd	CACACCATGTGTGAAGAGGAGACC	61	54
ACTG2 cDNA Rev	TCTGACTTTAGAAGCACTTCCTGTGGAC	62	46
IRES cDNA Fwd	CTCCCCCCCCCCTAACGTTA	61	65
IRES cDNA Rev	ATGTGTGGCCATATTATCATCGTGTTTTTC	60	37
tdRFP cDNA Fwd	ATGGTGGCCTCCTCCGAGGA	64	65
tdRFP cDNA Rev	CTACAGGAACAGGTGGTGGCGG	63	64

Supplementary Table 2. RT-qPCR Primers

**Primer Name**	**Sequence 5' → 3'**	**T_m (ºC)**	**GC (%)**
YWHAZ Fwd	TGCTTGCATCCCACAGACTA	57	50
YWHAZ Rev	AGGCAGACAATGACAGACCA	57	50
ACTG2 3' Endo/Exo Fwd	GTCCACAGGAAGTGCTTCTAAAGT	58	46
ACTG2 3' endo Rev	TGTTTCATGACTGGTAACAGAGTAGTC	58	41
IRES Rev qPCR	CTTATTCCAAGCGGCTTCGG	57	55
ACTG2 Fwd	CATGTACGTCGCCATTCAAGC	58	52
ACTG2 Rev	TTGATGTCTCGCACAATTTCTCT	56	39
ACTA2 Fwd	CACTGTCAGGAATCCTGTGA	55	50
ACTA2 Rev	CAAAGCCGGCCTTACAGA	56	56
MYH11 Fwd	AGATGGTTCTGAGGAGGAAACG	58	50
MYH11 Rev	AAAACTGTAGAAAGTTGCTTATTCACT	55	30

Supplementary Table 3. Antibodies

Antibody Name (species)	Application (Concentration)	Manufacturer	Catalog
Alexa Fluor 488 Phalloidin	Immunofluorescence (1:50)	Invitrogen	A12379 (RRID not listed)
Alexa Fluor 647 Phalloidin	Immunofluorescence (1:50)	Invitrogen	A22287 RRID:AB_2620155
Hoechst 33342, Trihydrochloride, Trihydrate	Immunofluorescence (1:1000)	Invitrogen	H3570
Pan-Actin (Rabbit)	Western Blot (1:1000)	Cytoskeleton, Inc.	AAN01 RRID:AB_10708070
V5 (rabbit)	Immunofluorescence (1:200)<br>Western Blot (1:1000)	Cell Signaling Technology	D3H8Q RRID:AB_2687461
V5 (mouse)	Immunofluorescence (1:100)	Abcam	ab53418 RRID:AB_883403
FLAG M2 (Mouse)	Immunofluorescence (1:200)	Sigma-Aldrich	F3165 RRID:AB_259529
MRTF-A (MKL1) (Rabbit)	Immunofluorescence (1:100)	Abcam	ab49311 RRID:AB_2235171
Smooth Muscle Myosin Heavy Chain (SMMS-1) (mouse)	Immunofluorescence (1:100)	Abcam	ab106919 RRID:AB_10866244
Calponin (CNN1) (mouse)	Immunofluorescence (1:10,000)	Sigma-Aldrich	C2687 RRID:AB_476840
Smooth Muscle 22α (TAGLN) (Rabbit)	Immunofluorescence (1:500)	Abcam	ab14106 RRID:AB_443021
Donkey anti-Rabbit IgG (H+L) Highly Cross-Adsorbed Secondary Antibody, Alexa Fluor 647	Immunofluorescence (1:400)	Life Technologies	A-31573 RRID:AB_2536183
Donkey anti-Rabbit IgG (H+L) Highly Cross-Adsorbed Secondary Antibody, Alexa Fluor 594	Immunofluorescence (1:400)	Life Technologies	A-21207 RRID:AB_141637
Donkey anti-Rabbit IgG (H+L) Highly Cross-Adsorbed Secondary Antibody, Alexa Fluor 555	Immunofluorescence (1:400)	Life Technologies	A-31572 RRID:AB_162543
Donkey anti-Mouse IgG (H+L) Highly Cross-Adsorbed Secondary Antibody, Alexa Fluor 488	Immunofluorescence (1:400)	Life Technologies	A-21202 RRID:AB_141607
Donkey anti-Mouse IgG (H+L) Secondary Antibody, Alexa Fluor 594	Immunofluorescence (1:400)	Life Technologies	A21203 RRID:AB_141633
Donkey anti-Mouse IgG (H+L) Secondary Antibody, Alexa Fluor® 647 conjugate	Immunofluorescence (1:400)	Life Technologies	A-31571 RRID:AB_162542
IRDye® 680RD Donkey anti-Mouse IgG Secondary Antibody	Western Blot (1:15,000)	Li-Cor	926-68072 RRID:AB_10953628
IRDye® 800CW Donkey anti-Rabbit IgG Secondary Antibody	Western Blot (1:15,000)	Li-Cor	926-32213 RRID:AB_621848

Supplementary Data 1. Imaris Analysis Parameters for Filament Analysis

V5 Filaments

<creationparameter classtypeid="bpFilament" name="Filaments - V5">

<bpFilamentCreateParametersThreshold mFormatVersion="8.0" mStep="eAlgorithm" mEnableRegionsOfInterest="false" mEnableRegionsOfInterestAllTimePoints="false" mProcessingType="eEntireImage" mAlgorithmType="eThreshold" mSourceChannelIndex="4" mBuildAllTimePoints="true" mTrack="false" mTrackDendrite="true" mTrackSpine="true" mTrackGraphMaxDist="-1" mTrackGraphGapsClose="true" mTrackGraphGapsSize="2" mDendriteBranchPointsTrackEnabled="true" mDendriteBranchPointsMaxDistance="-1" mDendriteBranchPointsGapClose="false" mDendriteBranchPointsGapSize="2" mDendriteTerminalPointsTrackEnabled="true" mDendriteTerminalPointsMaxDistance="-1" mDendriteTerminalPointsGapClose="false" mDendriteTerminalPointsGapSize="2" mSpineAttachmentPointsTrackEnabled="true" mSpineAttachmentPointsMaxDistance="-1" mSpineAttachmentPointsGapClose="false" mSpineAttachmentPointsGapSize="2" mSpineBranchPointsTrackEnabled="true" mSpineBranchPointsMaxDistance="-1" mSpineBranchPointsGapClose="false" mSpineBranchPointsGapSize="2" mSpineTerminalPointsTrackEnabled="true" mSpineTerminalPointsMaxDistance="-1" mSpineTerminalPointsGapClose="false" mSpineTerminalPointsGapSize="2" mFilterType="eOff" mFilterGaussianWidth="0.333496" mFilterEdgePreservingWidth="0.333496" mConnectiveBaseline="false" mThresholdLow="0" mThresholdHigh="10" mFillHoles="false" mBranchLengthMinLimit="1.5" mFindBeginningPoint="false" mTimePoint="0" mDeleteWorkingChannel="true"><bpRegionOfInterestContainer/></bpFilamentCreateParametersThreshold>

Phalloidin Filaments

<creationparameter classtypeid="bpFilament" name="Filaments - Phalloidin">

<bpFilamentCreateParametersThreshold mFormatVersion="8.0" mStep="eAlgorithm"
mEnableRegionsOfInterest="false" mEnableRegionsOfInterestAllTimePoints="false"
mProcessingType="eEntireImage" mAlgorithmType="eThreshold" mSourceChannelIndex="5"
mBuildAllTimePoints="true" mTrack="false" mTrackDendrite="true" mTrackSpine="true"
mTrackGraphMaxDist="-1" mTrackGraphGapsClose="true" mTrackGraphGapsSize="2"
mDendriteBranchPointsTrackEnabled="true" mDendriteBranchPointsMaxDistance="-1"
mDendriteBranchPointsGapClose="false" mDendriteBranchPointsGapSize="2"
mDendriteTerminalPointsTrackEnabled="true" mDendriteTerminalPointsMaxDistance="-1"
mDendriteTerminalPointsGapClose="false" mDendriteTerminalPointsGapSize="2"
mSpineAttachmentPointsTrackEnabled="true" mSpineAttachmentPointsMaxDistance="-1"
mSpineAttachmentPointsGapClose="false" mSpineAttachmentPointsGapSize="2"
mSpineBranchPointsTrackEnabled="true" mSpineBranchPointsMaxDistance="-1"
mSpineBranchPointsGapClose="false" mSpineBranchPointsGapSize="2"
mSpineTerminalPointsTrackEnabled="true" mSpineTerminalPointsMaxDistance="-1"
mSpineTerminalPointsGapClose="false" mSpineTerminalPointsGapSize="2"
mFilterType="eOff" mFilterGaussianWidth="0.333496" mFilterEdgePreservingWidth="0.333496"
mConnectiveBaseline="false" mThresholdLow="0" mThresholdHigh="10" mFillHoles="false"
mBranchLengthMinLimit="1.5" mFindBeginningPoint="false" mTimePoint="0"
mDeleteWorkingChannel="true">
<bpRegionOfInterestContainer>
</bpRegionOfInterestContainer>
</bpFilamentCreateParametersThreshold>

Supplementary Data 2. Imaris Analysis Parameters for Cell Migration Analysis


```
  <creationparameter classtypeid="bpPointsViewer" name="Nuclear tdRFP Spots Live
Imaging">
<bpPointsCreationParameters mFormatVersion="9.0" mEnableRegionOfInterest="false"
mEnableRegionGrowing="false" mEnableTracking="true" mProcessEntireImage="false"
mSourceChannelIndex="0" mEstimatedDiameter="10.000 10.000 10.000"
mBackgroundSubtraction="false" mRegionGrowingType="eLocalContrast"
mRegionGrowingAutomaticTreshold="true" mRegionGrowingManualThreshold="0"
mRegionGrowingDiameter="eDiameterFromVolume" mCreateRegionChannel="false">
<bpRegionOfInterestContainer>
</bpRegionOfInterestContainer>
<mSpotFilter>
<bpStatisticsFilter mLowerThresholdEnable="true" mLowerThresholdManual="true"
mLowerThresholdManualInitToAuto="false" mLowerThresholdManualValue="4749"
mUpperThresholdEnable="false" mUpperThresholdManual="false"
mUpperThresholdManualInitToAuto="false" mUpperThresholdManualValue="1"
mSelectHigh="true" mManualThreshold="true" mManualThresholdValue="4749.000"
mInitManualThresholdToAuto="false">
  <bpStatisticsValueType mName="Quality" mUnit="" mFactors="0"/>
</bpStatisticsFilter>
</mSpotFilter>
<bpObjectTrackingAlgorithmParameters TrackAlgoName="Brownian Motion"
mFillGapEnable="true" mReferenceFramesId="0">
<ObjectTrackingAlgorithmLinearAsignment MaxGapSize="3" MaxDistance="40"/>
</bpObjectTrackingAlgorithmParameters>
<mTrackFilter>
```

<bpStatisticsFilter mLowerThresholdEnable="true" mLowerThresholdManual="true" mLowerThresholdManualInitToAuto="false" mLowerThresholdManualValue="748.171" mUpperThresholdEnable="false" mUpperThresholdManual="false" mUpperThresholdManualInitToAuto="false" mUpperThresholdManualValue="1" mSelectHigh="true" mManualThreshold="true" mManualThresholdValue="748.171" mInitManualThresholdToAuto="false">
  <bpStatisticsValueType mName="Track Duration" mUnit="s" mFactors="0"/>
</bpStatisticsFilter>
</mTrackFilter>
</bpPointsCreationParameters>

References (all other references are provided in the main text)

32. Pettersen EF et al. UCSF Chimera--a visualization system for exploratory research and analysis. *J Comput Chem.* 2004;25:1605–1612.

33. Svitkina T. Imaging Cytoskeleton Components by Electron Microscopy. *Methods Mol Biol.* 2016;1365:99–118.
